# Comprehensive characterization of SLC41A3 identifies it as an immune-related prognostic biomarker and therapeutic target in hepatocellular carcinoma

**DOI:** 10.3389/fimmu.2026.1861310

**Published:** 2026-06-03

**Authors:** Biyan Gong, Dunfu Cao, Shidong Hu, Xiangyi Zhou, Leida Zhang, Fengsheng Dai, Jianheng Peng

**Affiliations:** 1 Department of Hepatobiliary Surgery, The First Affiliated Hospital (Southwest Hospital) of Army Medical University, Chongqing, China; 2 Department of General Surgery, Armed Police Corps Hospital, Chongqing, China; 3 Department of Spine Surgery, Zhongda Hospital Southeast University, Nanjing, China; 4 Chongqing Key Laboratory for the Mechanism and Intervention of Cancer Metastasis, Chongqing University Cancer Hospital, Chongqing University, Chongqing, China; 5 Health Management Center, The First Affiliated Hospital of Chongqing Medical University, Chongqing, China

**Keywords:** hepatocellular carcinoma, immune infiltration, prognostic biomarker, SLC41A3, tumor microenvironment

## Abstract

**Background:**

Hepatocellular carcinoma (HCC) represents a highly aggressive cancer associated with substantial patient mortality. Members of the solute carrier (SLC) protein family have been implicated in facilitating tumor development and progression. However, the specific functions and relevance of SLC41A3 in the context of HCC pathogenesis are not well defined.

**Methods:**

Differentially expressed SLC genes were screened from the TCGA-LIHC cohort. Prognostic analysis identified independent prognostic factors. Diagnostic and prognostic models were constructed using machine learning and LASSO regression. Multi-omics analysis was employed to assess the expression characteristics, clinical relevance, spatial heterogeneity, and correlation with immune infiltration of SLC41A3. The biological function of SLC41A3 was validated through *in vitro* proliferation, colony formation, migration, and invasion assays, as well as an *in vivo* xenograft tumor model.

**Results:**

Sixty-nine SLC genes were differentially expressed in HCC, with eight identified as independent prognostic factors, including SLC41A3. The constructed diagnostic and prognostic models based on these genes demonstrated robust performance. SLC41A3 was highlighted as the most contributory feature in the diagnostic model. High expression of SLC41A3 was significantly associated with advanced clinicopathological features, poorer patient survival, specific spatial expression patterns, and altered immune cell infiltration in the tumor microenvironment. Functionally, knockdown of SLC41A3 significantly inhibited HCC cell proliferation, migration, invasion *in vitro*, and suppressed tumor growth *in vivo*.

**Conclusion:**

This study identifies SLC41A3 as a novel prognostic biomarker and a promoter of HCC progression, with its function potentially linked to immune microenvironment modulation, suggesting its promise as a therapeutic target for HCC.

## 1 Introduction

Hepatocellular carcinoma (HCC) stands as a major contributor to cancer-associated deaths globally, a high lethality largely driven by its intricate tumor heterogeneity, metastatic potential, and constrained treatment efficacy (1–3). The advancement of tumors is governed not merely by the intrinsic malignant activities of cancer cells, including rampant proliferation and invasive capacity, but is also significantly influenced by the surrounding tumor microenvironment (TME) (4–7). A pivotal factor within the TME that shapes clinical outcomes and therapeutic efficacy is the landscape of infiltrating immune cells. Consequently, discovering and characterizing pivotal molecules that orchestrate both the aggressive intrinsic behavior of HCC cells and the remodeling of the immune milieu is of paramount importance for identifying new prognostic indicators and actionable therapeutic targets.

The solute carrier (SLC) superfamily encompasses numerous membrane-bound transporter proteins that fulfill critical functions in shuttling diverse substrates across cellular membranes, thereby maintaining metabolic equilibrium and facilitating intracellular signaling (8–10). Accumulating data suggest that distinct SLC proteins significantly contribute to oncogenic processes, including metabolic reprogramming, the development of drug resistance, and immune modulation. Within this extensive family, solute carrier family 41 member 3 (SLC41A3) represents a relatively uncharacterized protein whose complete functional profile, clinical relevance, and mechanistic roles in malignancies, especially HCC, remain largely undefined (11). Key unresolved questions include the prognostic potential of SLC41A3 in HCC, its interplay with the tumor immune landscape, and its direct influence on driving malignant progression, all of which warrant urgent investigation.

Therefore, this study was designed to comprehensively investigate the clinical relevance, biological functions, and interactions with the tumor immune microenvironment of SLC41A3 in HCC. Our investigation commenced with an analysis of the TCGA-LIHC cohort, which identified 69 SLC family genes displaying differential expression in HCC. Subsequent survival analysis pinpointed 8 of these genes as independent factors significantly correlated with patient overall survival. Utilizing these 8 SLC genes, we developed diagnostic and prognostic risk models employing machine learning and LASSO regression techniques, respectively. Both models exhibited robust diagnostic capability and prognostic predictive accuracy within the training cohort and an independent validation set. Notably, interpretability analysis (SHAP) indicated that SLC41A3 was the most influential feature in the diagnostic model. Furthermore, enrichment analysis of genes differentially expressed between prognostic risk groups implied a potential role for SLC41A3 in modulating the immune microenvironment. These findings led us to select SLC41A3 for focused exploration. We subsequently conducted an extensive multi-omics analysis to delineate the expression patterns, clinical prognostic significance, spatial heterogeneity, and correlations with immune infiltration of SLC41A3 in HCC. Finally, employing both *in vitro* and *in vivo* experimental approaches, we empirically validated the role of SLC41A3 in promoting HCC cell proliferation, migration, invasion, and tumorigenic potential. This work provides the first systematic evidence positioning SLC41A3 as a crucial prognostic biomarker and oncogenic driver in HCC, while preliminarily uncovering its association with the tumor immune landscape, thereby offering novel perspectives for HCC prognosis evaluation and targeted therapeutic strategies.

## 2 Materials and methods

### 2.1 Data acquisition and processing

This study utilized the TCGA-LIHC (Liver Hepatocellular Carcinoma) dataset as the training and internal validation set (via 7:3 random split). Gene expression profiles and clinical data for TCGA-LIHC were downloaded from the UCSC Xena database. The gene list for the SLC family was obtained from the Gene Cards. Differential expression analysis between tumor and adjacent normal tissues in TCGA-LIHC, as well as between high- and low-risk groups defined by the prognostic model, was performed using the R package limma (version 3.58.1). Relevant plots were visualized using the ggplot2 package (version 4.0.2).

### 2.2 Machine learning model construction

To build a robust prognostic prediction model, ten classical machine learning algorithms were selected. Using the TCGA-LIHC dataset as the training set, the SLC-based risk score and key clinical features served as input variables. The caret package (version 7.0.1) was employed for unified model training and hyperparameter tuning. The model’s generalizability was evaluated in the independent GSE244826 validation set. Model performance was comprehensively assessed from three aspects: 1) Discriminative Ability: Evaluated by plotting Receiver Operating Characteristic (ROC) curves and calculating the Area Under the Curve (AUC). Statistical analysis was performed using the pROC package (version 1.19.0.1). 2) Clinical Utility: Assessed using Decision Curve Analysis (DCA) to determine the clinical net benefit at different threshold probabilities, implemented with the rmda package (version 1.6). 3)Interpretability: Based on SHapley Additive exPlanations (SHAP) values, the kernelshap (version 0.9.1) and shapviz (version 0.10.3) packages were used to analyze the importance and directional contribution of each feature to the model’s predictions.

### 2.3 Prognostic signature construction

The analytical workflow for developing and validating the prognostic signature proceeded as follows. First, the TCGA-LIHC cohort was randomly partitioned into a training set and an internal validation set at a 7:3 ratio. Within the training set, genes belonging to the SLC transporter family that were differentially expressed between tumor and adjacent non-tumor tissues were identified. To initially screen for genes associated with patient survival, univariate Cox proportional hazards regression was performed. Subsequently, a stepwise multivariate Cox regression analysis was applied to refine the selection and identify a set of eight SLC genes demonstrating independent prognostic value. To enhance model generalizability and mitigate overfitting, a Least Absolute Shrinkage and Selection Operator (LASSO) regression analysis was implemented on the expression data of these eight genes using the glmnet package (v4.1.10). Genes retaining non-zero coefficients were selected for final model inclusion. A risk score for each patient was then calculated by multiplying the expression level of each selected gene by its corresponding LASSO-derived coefficient and summing the products. Using the median risk score from the training set as a cutoff, patients were categorized into high-risk and low-risk subgroups. The prognostic performance of the model was evaluated in both the training and validation sets. Kaplan-Meier survival curves with log-rank tests were used to assess the stratification ability of the risk groups. Furthermore, the time-dependent predictive accuracy of the risk score for overall survival at 1, 3, and 5 years was quantified by calculating the area under the receiver operating characteristic (ROC) curve using the time ROC package (v0.4). All statistical analyses and visualizations were conducted in the R environment, utilizing packages including survival (v3.6.4) and ggplot2 (v4.0.2).

### 2.4 Drug sensitivity analysis

Based on the gene expression profiles of samples in the training set, we employed the oncoPredict R package (version 1.2) to infer sensitivity to various drugs contained in the GDSC2 database and to estimate the corresponding half-maximal inhibitory concentration (IC_50_) values. Spearman correlation analysis was used to assess the association between the IC_50_ value of each drug and the patient’s risk score. To further explore potential differences in drug sensitivity between risk groups, we separately identified representative drugs showing the strongest positive correlation and the strongest negative correlation with the risk score for visualization. Plotting was done using the ggplot2 package (version 4.0.2).

### 2.5 Analysis of somatic mutations and the tumor immune microenvironment

To analyze somatic mutations, data from the TCGA-LIHC training and validation cohorts were processed using the maftools package (version 2.18.0). This allowed for the characterization of mutation types and frequencies, as well as the calculation and subsequent comparison of Tumor Mutational Burden (TMB) between patients categorized into high- and low-risk groups. Two complementary computational approaches were employed to characterize features of the tumor immune microenvironment. First, the ESTIMATE package (version 1.0.13) was utilized to compute immune and stromal scores, along with an estimation of tumor purity. Second, applying the ssGSEA (single-sample Gene Set Enrichment Analysis) algorithm via the GSVA package (version 1.50.5), we assessed the relative infiltration levels of various immune cell populations and the activity scores of immune-related functional pathways. Additionally, the Wilcoxon rank-sum test was used to compare differences in the expression of genes related to Major Histocompatibility Complex (MHC) molecules, chemokines, and key cancer-related pathways between the high- and low-risk groups (adjusted FDR < 0.05).

### 2.6 Processing of spatial transcriptomic data

Spatial transcriptomics data were derived from a publicly available study on HCC (12). Analysis of the HCC section was conducted using the Sparkle system (https://www.grswsci.top).

### 2.7 Cell culture and generation of stable cell lines

The human HCC cell lines Hep3B and Huh7 were purchased from Procell Biotechnology. Cells were cultured in RPMI-1640 medium supplemented with 10% fetal bovine serum (FBS) and 1% penicillin/streptomycin at 37 °C in a humidified incubator with 5% CO_2_. All cell lines were authenticated by short tandem repeat (STR) profiling. Lentiviral vectors expressing short hairpin RNA (shRNA) targeting SLC41A3 and a non-targeting control (sh-NC) were purchased from Genechem Co., Ltd. (Shanghai, China). The specific shRNA sequences were:

sh-SLC41A3#1: 5’AAAAGCATGCTTCTGGACTATTTTTGGATCCAAAAATAGTCCAGAAGCATGC-3’,

sh-SLC41A3#2:

5’AAAAGGGTCAACCCAGACAACATTTGGATCCAAATGTTGTCTGGGTTGACCC-3’.

Hep3B and HuH7 cells were plated in 6-well plates and transduced at approximately 20-30% confluence. After 72 hours of incubation, stable integrants were selected with puromycin (1 μg/mL) for one week. Successful knockdown was confirmed by Western blotting in both cell lines.

### 2.8 Western blotting

Western blotting was performed according to previously described methods (13, 14). The antibodies used in this study included: rabbit monoclonal anti-SLC41A3 (GeneTex, GTX46859; 1:1000), mouse monoclonal anti-β-actin (Proteintech, 66009-1-Ig; 1:10,000), HRP-labeled goat anti-mouse secondary antibody (Proteintech, RGAM001; 1:10,000), and HRP-labeled goat anti-rabbit secondary antibody (Proteintech, RGAR001; 1:10,000).

### 2.9 Assessment of cell proliferation

Cell proliferation was evaluated using two complementary assays. For the CCK-8 assay, stable Hep3B and Huh7 cell lines were plated in 96-well plates. Cell viability was assessed at designated time points (0, 24, 48, 72, and 96 hours) following the addition of 10 μl CCK-8 solution per well and subsequent incubation in darkness for two hours. Each experimental condition was assayed in triplicate. In parallel, a colony formation assay was performed. Cells were seeded onto 6-well plates and maintained in culture for a period of 14 days, with the culture medium being refreshed every seven days. Upon completion of the incubation period, the resulting cell colonies were fixed using 4% paraformaldehyde, stained with a 0.5% crystal violet solution, and then subjected to imaging and manual counting. This experiment was conducted in three independent biological replicates.

### 2.10 Migration and invasion analysis

To evaluate two-dimensional migration, a wound healing assay was conducted. Hep3B and Huh7 cells were cultured in 6-well plates until reaching complete confluence. Following a 12-hour period of serum deprivation, a uniform linear wound was introduced across the cell monolayer using a sterile pipette tip. After washing to remove detached cells, the cultures were maintained in medium supplemented with 2% FBS. Images of the wound area were captured immediately after scratching (0 hour) and after 24 hours of incubation. The extent of wound closure was quantified using ImageJ software. This experiment was performed with three technical replicates per condition. For the quantitative assessment of migration and invasion through a membrane, Transwell chamber assays were employed. For the migration assay, cells were seeded into the upper chamber of Transwell inserts without any matrix coating. To evaluate invasive potential, the inserts were pre-coated with a layer of Matrigel basement membrane matrix. In both setups, cells suspended in serum-free medium were placed in the upper compartment, while the lower chamber was filled with medium containing 20% FBS as a chemoattractant. Following an appropriate incubation period, cells that had traversed the membrane were fixed, stained with crystal violet, and manually counted under an inverted microscope. All Transwell experiments were conducted in triplicate.

### 2.11 *In vivo* subcutaneous tumor model

Animal studies were approved by the Animal Ethics Committee of Laboratory Animal Welfare and Ethics Committee of the Third Military Medical University (Approval No. AMUWEC20224100). The following humane endpoints were predetermined to ensure animal welfare: (1) tumor volume exceeding 1500 mm³; (2) ulceration or necrosis of the tumor; (3) body weight loss exceeding 20% of the initial weight; (4) signs of severe distress or impaired mobility. Three 4-week-old female nude mice were housed under specific pathogen-free conditions. Each mouse received bilateral subcutaneous injections of Hep3B cells (transduced with sh-SLC41A3#1 or sh-NC) into the axillary region. Tumor size was measured every other day starting from day 7 post-injection, and volume was calculated. Throughout the study, no animals reached these predefined endpoints before the scheduled termination. On day 23, mice were euthanized by gradual displacement of chamber air with compressed CO_2_ at a flow rate of 30-40% of the chamber volume per minute, followed by confirmation of death by cervical dislocation. Tumors were then harvested and weighed.

### 2.12 Analysis of immune cell infiltration

The relative abundances of 22 immune cell types in the tumor microenvironment were estimated using the CIBERSORT algorithm with the LM22 signature matrix (15). This method was selected for its widespread use and validation in transcriptomic deconvolution studies of cancer.

### 2.13 Statistical analysis

All data processing and statistical analyses in this study were performed using R language (version 4.5.1). The software packages and their core functions used in the main analytical workflows have been detailed in the corresponding sections above. These primarily included packages for differential expression analysis (limma), survival analysis (survival), model construction and regression (glmnet, rms), functional enrichment analysis (clusterProfiler, DOSE, enrichplot), genomic analysis (maftools), and tumor immune microenvironment analysis (estimate, GSVA). Unless otherwise specified, a two-sided P value < 0.05 was considered statistically significant. All graphics were generated and assembled using the ggplot2 (version 4.0.2) and cowplot (version 1.2.0) packages.

## 3 Results

### 3.1 Integrating SLC family genes via machine learning to construct diagnostic and prognostic models for HCC

**Figure 1:**
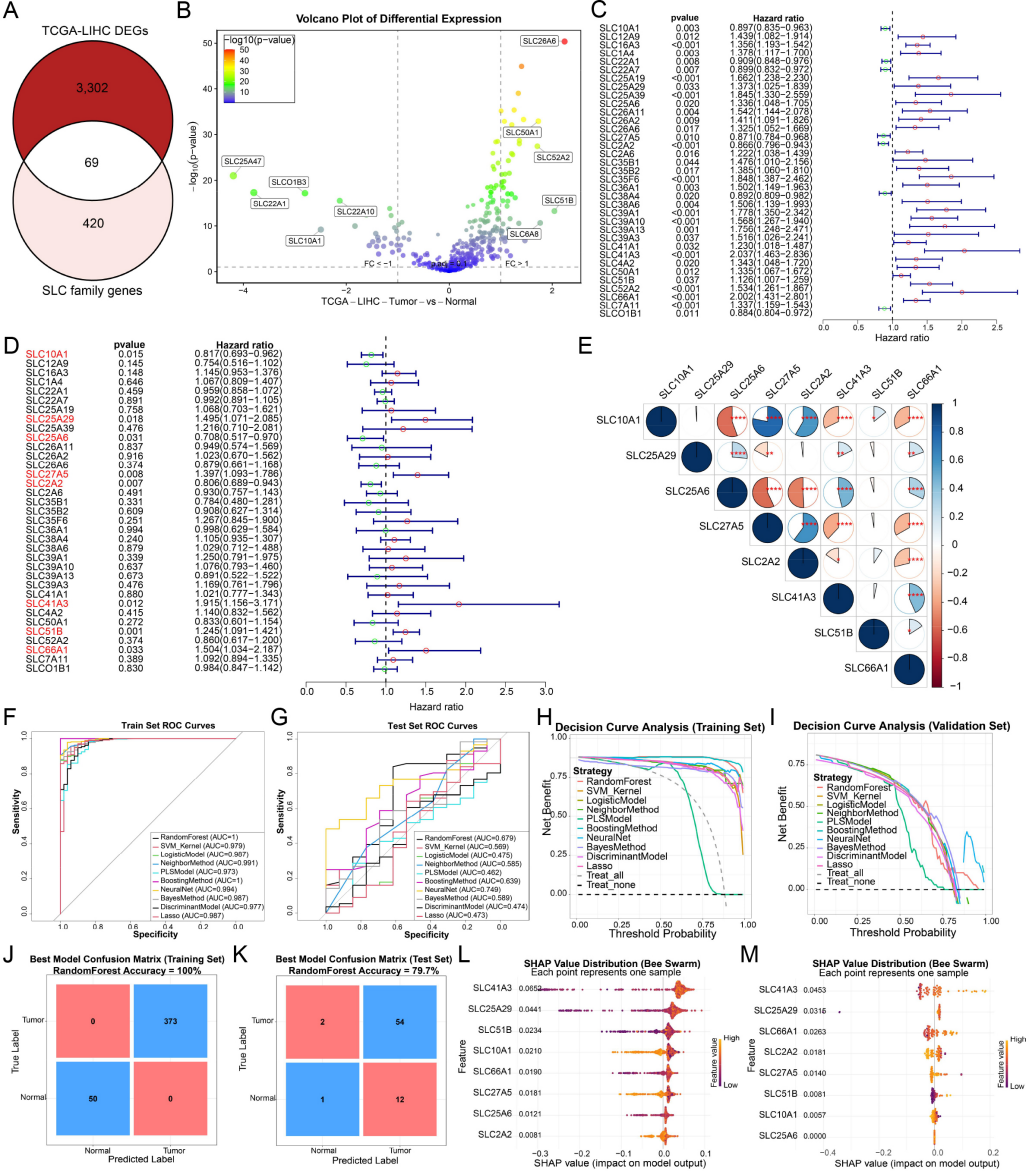
Identification of SLC family genes and construction of a prognostic model in hepatocellular carcinoma. **(A)** Venn diagram of candidate genes. **(B)** Volcano plot illustrating differentially expressed SLC family genes. **(C)** Univariate forest plot displaying 35 SLC genes with prognostic significance among the 69 differentially expressed genes. **(D)** Multivariate analysis identified the optimal set of 8 SLC genes associated with HCC prognosis. **(E)** Correlation heatmap of the 8 prognostic genes. **(F, G)** ROC curves of ten different models in the TCGA-LIHC training set **(F)** and the GEO (GSE244826) validation set **(G)**. **(H, I)** Decision curves of the ten models in the TCGA-LIHC training cohort **(H)** and the GEO validation set **(I)**. **(J, K)** Confusion matrices of the Random Forest model in the TCGA-LIHC training set **(J)** and the GEO test set **(K)**. **(L, M)** SHAP values for each feature across different levels in the Random Forest model for the TCGA-LIHC training cohort **(L)** and the GEO test set **(M)**. *P < 0.05, ** *P* < 0.01, *** *P* < 0.001.

The SLC family comprises crucial transmembrane transporter proteins, whose dysregulation is intimately associated with the pathogenesis and progression of HCC. To systematically investigate the diagnostic and prognostic value of SLC genes in HCC, we first performed differential expression analysis between tumor and adjacent normal tissues using RNA-seq data from the TCGA-LIHC cohort via the limma R package. Applying thresholds of |log2 fold change (FC)| > 1 and *P* value < 0.05, we identified 3,371 differentially expressed genes (DEGs). Intersection of these DEGs with the known SLC gene set revealed 69 differentially expressed SLC genes in HCC (**Figure 1A**). A volcano plot visually represents the expression differences of these SLC genes (**Figure 1B**).

To assess the prognostic significance of these differential SLC genes, we conducted prognostic modeling. After excluding samples with missing survival information, 364 patients were randomly divided into a training set (n=255) and a validation set (n=109) at a 7:3 ratio. Univariate (**Figure 1C**) and subsequent multivariate Cox regression analyses (**Figure 1D**) in the training set identified 8 SLC genes (SLC10A1, SLC25A29, SLC25A6, SLC27A5, SLC2A2, SLC41A3, SLC51B, and SLC66A1) as independent prognostic factors significantly associated with overall survival (OS). The expression correlations among these 8 genes across TCGA-LIHC samples are presented in a heatmap (**Figure 1E**). Consistent differential expression of these eight genes was observed at both the transcriptomic and proteomic levels in tumor tissues (**Supplementary Figures 1A–C**). Kaplan-Meier survival analysis further confirmed significant correlations between their expression levels and patient OS (**Supplementary Figure 1D**).

To evaluate the diagnostic potential of these eight SLC genes for HCC, we constructed ten machine learning models based on their expression profiles. In the TCGA training set, all models achieved an area under the ROC curve (AUC) greater than 0.5 (**Figure 1F**). In the independent validation set (GSE244826), the Random Forest and Neural Net models yielded the highest AUC values (**Figure 1G**). Decision curve analysis (DCA) indicated that all models provided favorable clinical net benefit in the training set (**Figure 1H**), whereas only a subset of models (Random Forest, Neural Net, and Bayes Method) demonstrated clinical utility in the validation set (**Figure 1I**). The Random Forest model was preliminarily identified as the optimal diagnostic model, with confusion matrices confirming its high diagnostic accuracy in both the training and validation sets (**Figures 1J, K**). SHAP value analysis was employed to interpret feature importance, revealing that SLC41A3 contributed most substantially to the model’s predictive power for distinguishing disease status in both cohorts (**Figures 1L, M**).

**Figure 2:**
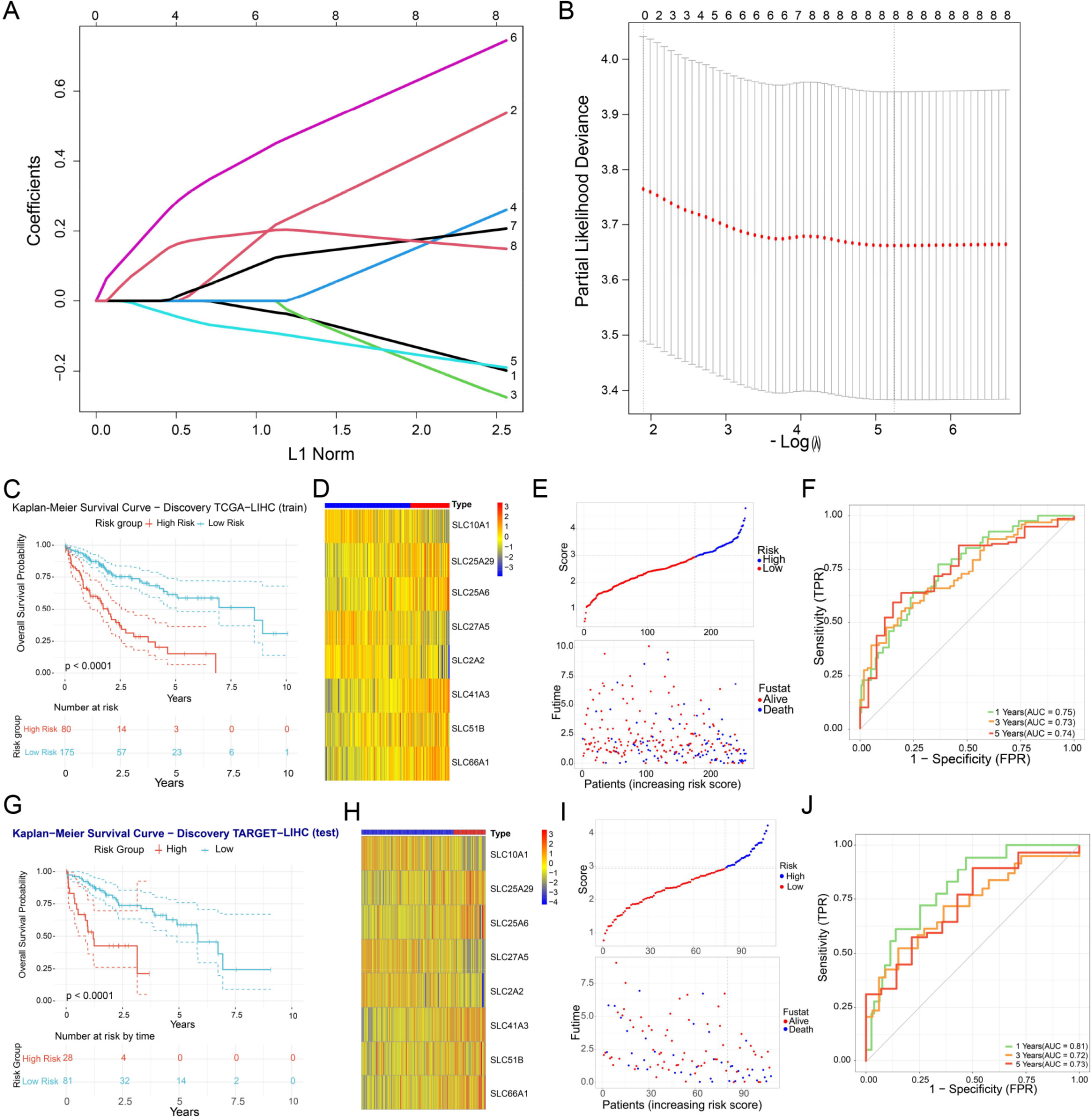
Construction and validation of the SLC-based prognostic risk model in HCC. **(A)** Trajectories of regression coefficients for eight differentially expressed SLC genes associated with patient prognosis, generated through LASSO regularization. **(B)** Eight genes were defined as independent prognostic determinants and incorporated into the risk model based on the optimal penalty parameter (lambda.min). **(C)** The training cohort was stratified into high- and low-risk subgroups, with their respective overall survival probabilities compared via Kaplan-Meier estimates. **(D)** A heatmap illustrates the expression patterns of the signature genes across the defined risk strata within the training set. **(E)** For patients in the training cohort, scatter plots display the calculated risk scores alongside individual survival outcomes, ordered by increasing risk. **(F)** Survival outcomes between the high- and low-risk categories in the independent validation cohort were assessed using Kaplan-Meier analysis. **(G)** Expression levels of the core genes in the validation set are visualized in a heatmap, organized according to the established risk stratification. **(H)** The validation cohort’s patient distribution, plotted by ascending risk score, is annotated with corresponding survival status. **(I)** In the training set, receiver operating characteristic (ROC) curves evaluate the model’s accuracy for predicting overall survival at one, three, and five years. **(J)** The predictive performance of the signature for one-, three-, and five-year overall survival was further validated by ROC analysis in the independent cohort.

To develop a more robust prognostic model, we constructed a LASSO regression model based on the aforementioned eight SLC genes. The optimal penalty parameter (λ) was determined to be 0.0043 through cross-validation (**Figures 2A, B**). A risk score for each patient was calculated using the coefficients derived from the Lasso model. Patients were subsequently stratified into high-risk and low-risk groups based on an optimal cut-off value. In the training set, Kaplan-Meier survival analysis revealed a significant disparity in survival outcomes between the two risk groups (**Figure 2C**). The distribution of risk scores, where red and blue dots represent deceased and surviving patients respectively, indicated a substantially higher mortality rate within the high-risk subgroup (**Figure 2D**). A heatmap demonstrated consistent upregulation of all eight signature genes in high-risk patients (**Figure 2E**). Time-dependent receiver operating characteristic (ROC) analysis for predicting 1-, 3-, and 5-year survival yielded area under the curve (AUC) values all exceeding 0.5, affirming the model’s strong prognostic discriminatory power (**Figure 2F**). The predictive efficacy of this prognostic signature was consistently replicated and validated in the independent validation cohort (**Figures 2G–J**).

We next sought to elucidate the potential biological mechanisms through which this SLC-based prognostic signature influences patient outcomes. Differential expression analysis was performed between the high- and low-risk groups in the validation set using the limma R package (*P* < 0.05). Subsequent functional enrichment analyses, including GO annotation, KEGG pathway analysis, and GSEA, were conducted on the identified differentially expressed genes. GO analysis revealed significant enrichment of immune-related terms across biological processes, cellular components, and molecular functions, such as immune receptor activity, immune response, regulation of T cell activation, and antigen binding. KEGG analysis similarly showed significant enrichment in pathways associated with cell adhesion molecules and fundamental immune system processes, including autoimmune thyroid disease (**Supplementary Figures 2A–D**). These findings suggest that the prognostic model may exert its influence by modulating the tumor immune microenvironment.

### 3.2 Exploring mutational profiles in high- and low-risk groups of the SLC-based prognostic signature

**Figure 3:**
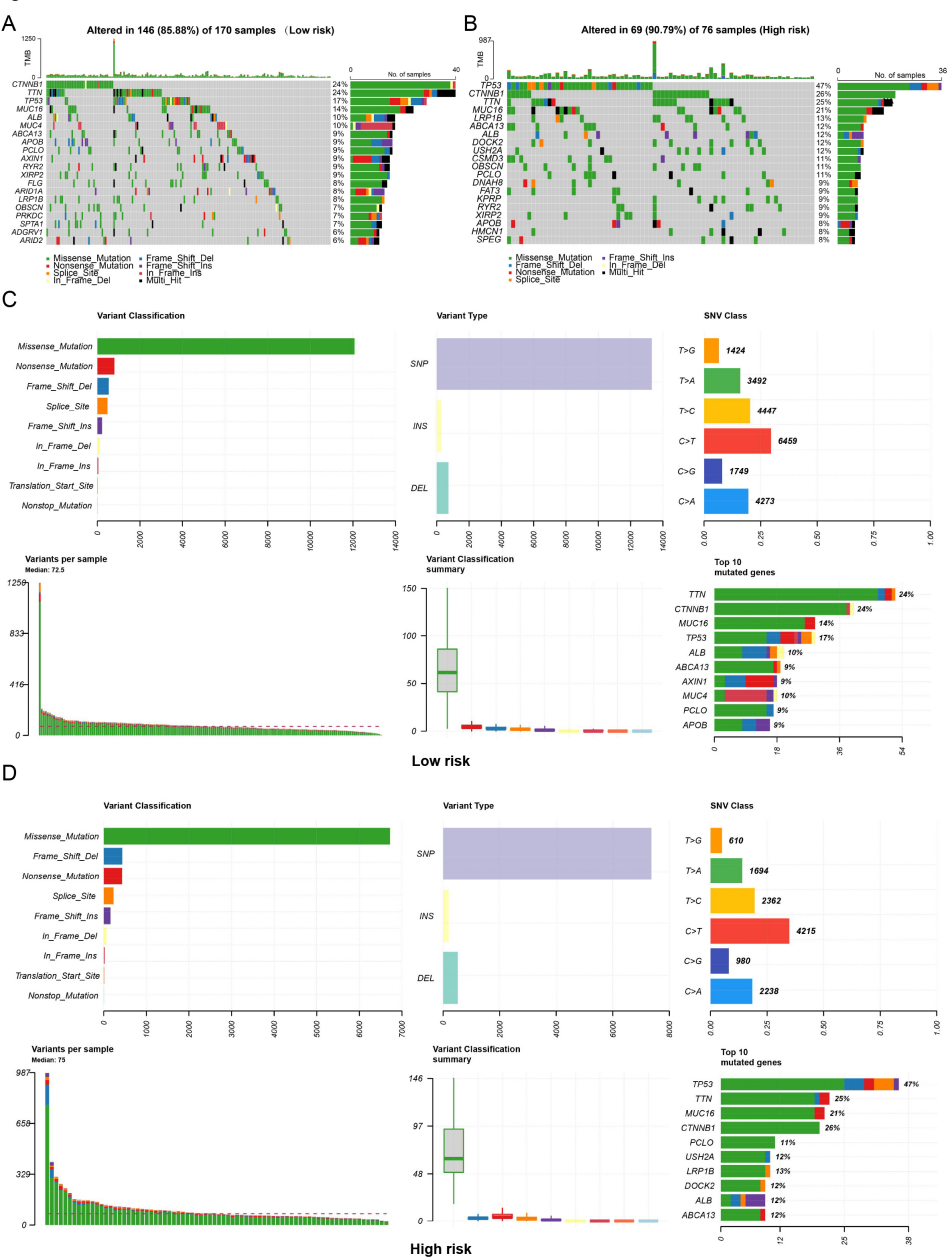
Assessment of tumor mutation burden across defined risk strata. **(A)** Waterfall plot depicting the somatic mutation spectrum for the most frequently mutated genes in patients stratified as low-risk. **(B)** A corresponding mutational landscape is presented for the cohort assigned to the high-risk category. **(C, D)** Comparative analysis of gene mutation frequencies **(C)** and overall tumor mutation burden **(D)** between the high- and low-risk groups.

Given the pronounced prognostic and functional enrichment disparities between the high- and low-risk groups defined by our SLC family-based prognostic model, we hypothesized that these distinctions might be linked to underlying genomic mutational features. To investigate this, we acquired and analyzed somatic mutation data from the TCGA-LIHC cohort. Mutational landscape plots revealed differences in mutation spectra between the two risk groups (**Figures 3A, B**). In the low-risk group, CTNNB1 and TTN exhibited the highest mutation frequencies, each reaching 24% (**Figure 3A**). Conversely, TP53 demonstrated a markedly elevated mutation frequency of 47% in the high-risk group (**Figure 3B**). Despite these differences in specific gene mutation rates, the predominant variant classification and type were consistent across both groups: the most common variant type was missense mutation within single nucleotide variants, and the most frequent nucleotide change was C>T transition (**Figures 3C, D**).

### 3.3 Immunological profiling reveals an immunosuppressive and tolerogenic microenvironment in high-risk HCC

**Figure 4:**
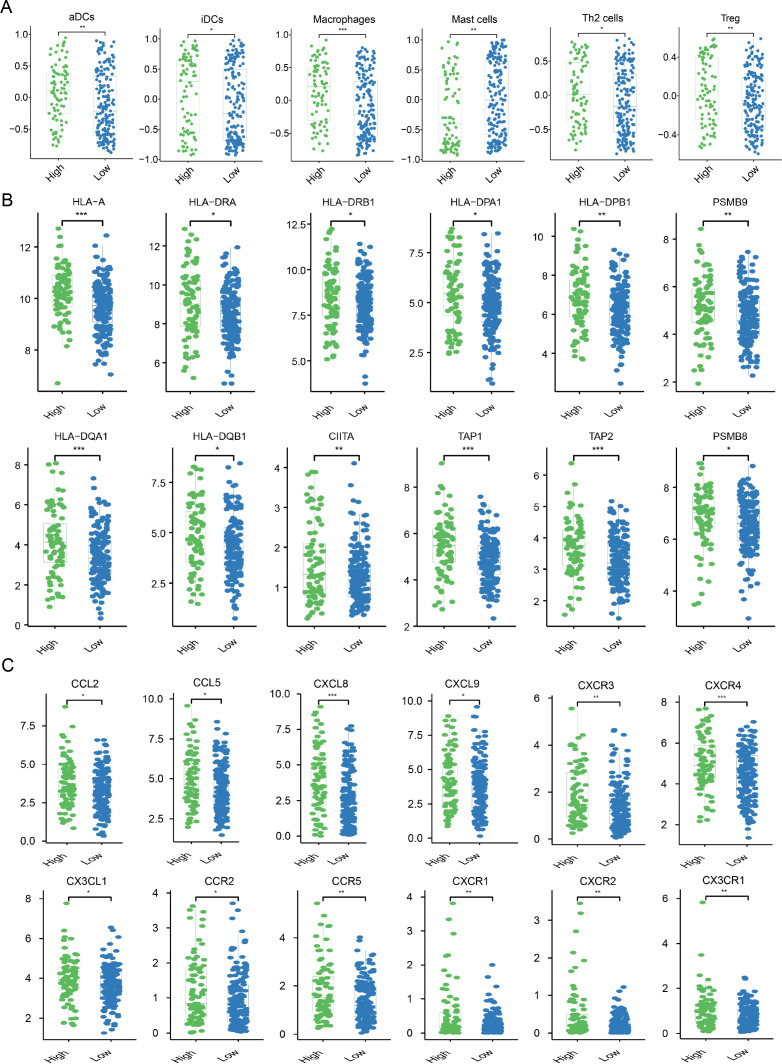
The high-risk HCC group exhibits an immunosuppressive tumor microenvironment. **(A)** Immune cell infiltration characteristics between the high- and low-risk groups analyzed based on the GSVA algorithm. Macrophages, regulatory T cells (Tregs), and Th2 cells were significantly activated in the high-risk group. **(B)** Heatmap showing the expression of major histocompatibility complex (MHC)-related genes between the high- and low-risk groups, indicating a partial enhancement of antigen presentation function. **(C)** Heatmap showing the expression of representative chemokines and their receptors between the high- and low-risk groups, with elevated expression in the high-risk group. Collectively, the results indicate that the tumor microenvironment of high-risk HCC exhibits an immunosuppressive state, characterized by the activation of pro-tolerogenic immune cells and upregulation of chemokine signaling, which may facilitate immune escape. *P < 0.05, ** *P* < 0.01, *** *P* < 0.001.

To further elucidate the immunological landscape distinguishing the prognostic groups, we conducted a comparative analysis of immune features between patients classified as high- and low-risk. Utilizing the GSVA algorithm, we quantified the infiltration landscape of diverse immune cell populations within each tumor sample. This profiling revealed substantial disparities in the abundance of several immune cell subsets. Notably, the high-risk cohort exhibited a marked activation state of macrophages, alongside significant enrichment of regulatory T cells (Tregs) and T helper 2 (Th2) cells (**Figure 4A**). Evaluation of Major Histocompatibility Complex (MHC) molecule expression indicated a modest upregulation in several antigen presentation-related functions (**Figure 4B**). These observations imply that while antigen presentation machinery may be partially intact, tumor cells in high-risk HCC potentially foster an immune-tolerant state by skewing the immune response towards a Th2/Treg-dominant axis. In parallel, we detected elevated expression levels of specific chemokines and their cognate receptors, including CCL2 and CCL5, within the high-risk group (**Figure 4C**), collectively pointing towards a functionally suppressed immune milieu.

### 3.4 Disparities in drug sensitivity between high- and low-risk groups stratified by the SLC-based prognostic signature

**Figure 5:**
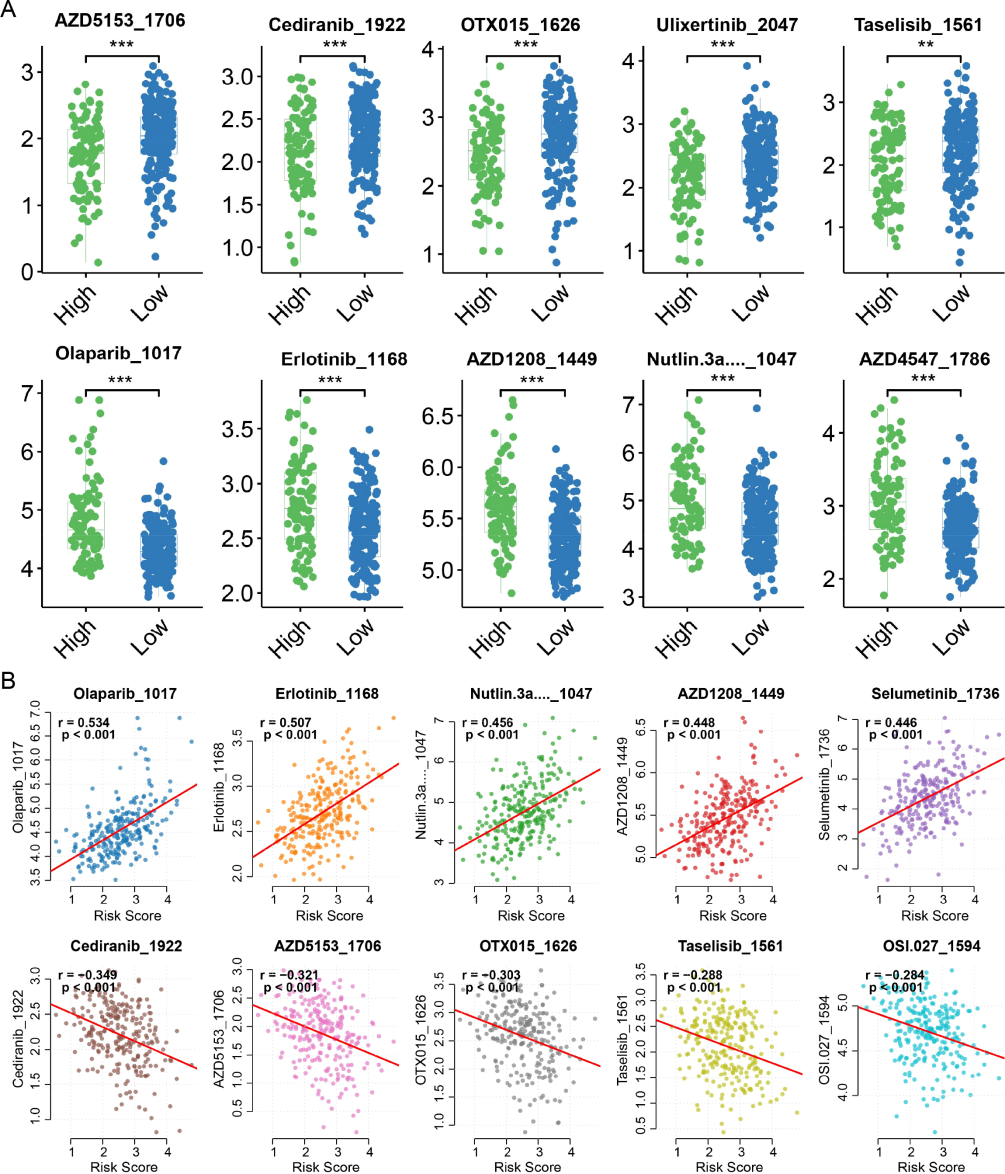
Drug sensitivity analysis. **(A)** Analysis of differences in IC50 values for the top five drugs showing the strongest negative and positive correlations with the risk score, between the two risk groups. **(B)** Spearman correlation analysis between the risk score and the IC50 values of the top five drugs with the strongest negative and positive correlations. *** *P* < 0.001.

To investigate the translational potential of the prognostic signature for informing therapeutic strategies, we conducted a systematic evaluation of drug sensitivity profiles. Utilizing the oncoPredict R package and leveraging the Genomics of Drug Sensitivity in Cancer (GDSC) database, we predicted the sensitivity (quantified as the half-maximal inhibitory concentration, IC50) of each patient sample to a panel of 545 compounds. Comparative analysis between the high-risk and low-risk cohorts identified ten pharmacological agents for which the predicted IC50 values differed significantly. The distribution of these IC50 values across the two groups was visualized using box plots (**Figure 5A**). Agents such as Olaparib demonstrated pronounced differential sensitivity between the risk groups, implying that risk stratification could potentially predict variable clinical responses to these therapies. Additionally, scatter plots were constructed to depict the correlation between the individual patient risk scores and the predicted IC50 values for each of these ten drugs (**Figure 5B**).

### 3.5 Multi-omics profiling, clinical prognostic value, and spatial expression pattern of SLC41A3 in HCC

Among the eight SLC genes constituting the prognostic signature, SLC41A3 was selected forin-depth investigation due to its highest hazard ratio, most significant statistical association,and previously undefined role in HCC. Comprehensive pan-cancer screening revealed that SLC41A3 was significantly upregulated in a variety of malignant tumors (**Supplementary Figures 3A–C**). Mutational profiling indicated that SLC41A3 had the highest alteration frequency inCervical Cancer (**Supplementary Figure 4A**). Copy number variation (CNV) analysis identified recurrent amplifications of SLC41A3 in Lung squamous cell carcinoma (LUSC), cervical squamous cell carcinoma and endocervical adenocarcinoma (CESC), and ovarian cancer (OV) (**Supplementary Figure 4B**). A significant positive correlation between SLC41A3 copy number and its mRNA expression wasobserved in breast invasive carcinoma (BRCA), pheochromocytoma and paraganglioma (PCPG), and LUSC(**Supplementary Figure 4C**). Furthermore, significant differences in SLC41A3 DNA hypermethylation levels were detectedacross multiple cancers, including LIHC, LUSC, and lung adenocarcinoma (LUAD) (**Supplementary Figure 5A**). Its methylation status showed a significant negative correlation with mRNA expression inuveal melanoma (UVM), PCPG, and kidney renal papillary cell carcinoma (KIRP) (**Supplementary Figure 5B**). These integrated multi-omics analyses collectively nominate SLC41A3 as a potential pan-cancer prognostic biomarker.

**Figure 6:**
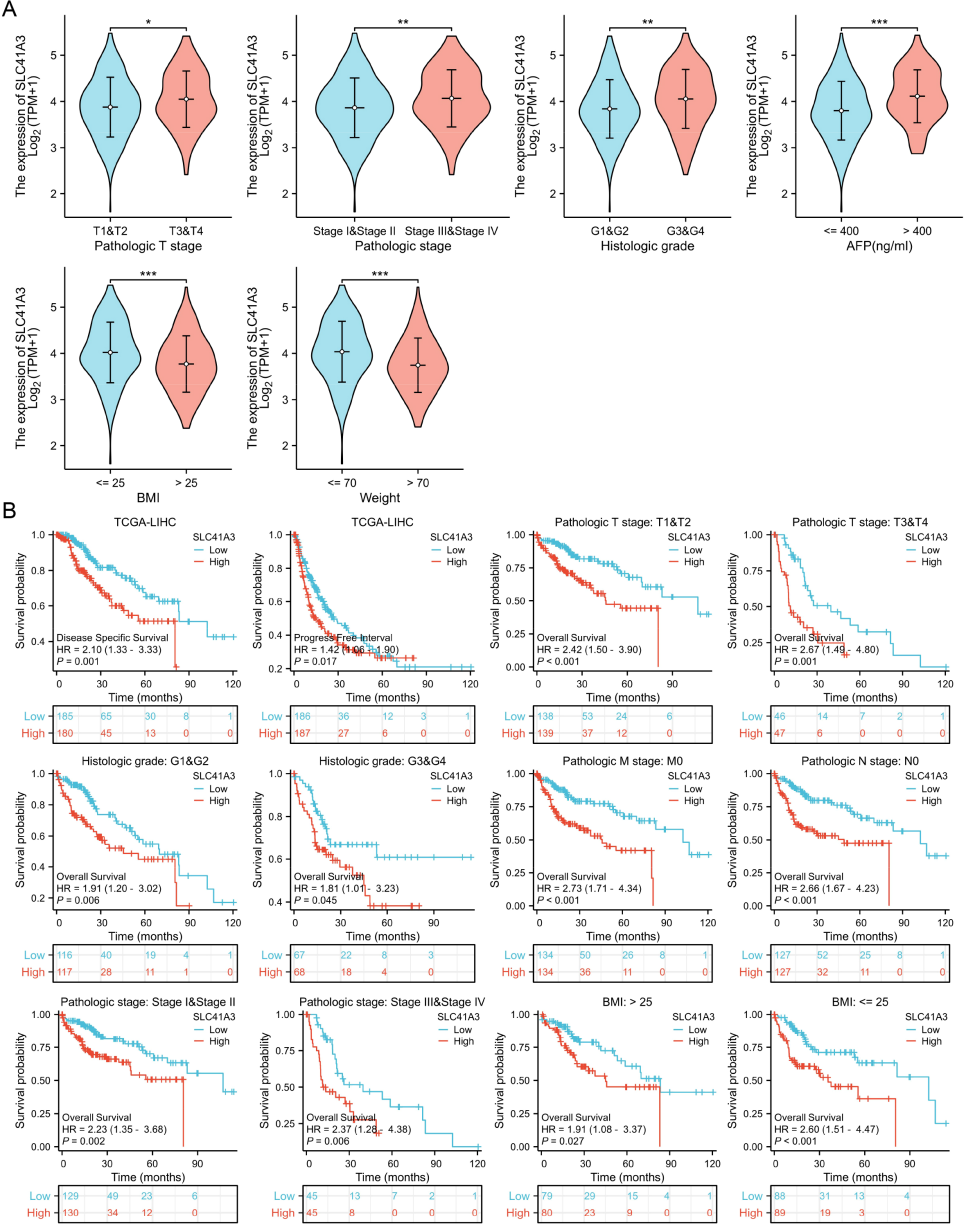
Association of SLC41A3 expression with clinicopathological features and prognosis in HCC patients. **(A)** Correlation between SLC41A3 expression levels and clinicopathological parameters in the TCGA-LIHC cohort. **(B)** Kaplan-Meier survival analysis evaluating the prognostic significance of SLC41A3 expression in TCGA-LIHC. * *P* < 0.05, ** *P* < 0.01, *** *P* < 0.001.

SLC41A3 expression was significantly correlated with several clinicopathological variables, including T stage, pathologic stage, histologic grade, alpha-fetoprotein (AFP) level, body mass index (BMI), and weight (**Figure 6A**). Survival analysis demonstrated that patients with high SLC41A3 expression had worse disease-specific survival (DSS) and progression-free interval (PFI). Further subgroup survival analysis revealed that high SLC41A3 expression was associated with poorer prognosis in key subgroups, such as those defined by T stage, histologic grade (G), absence of distant metastasis (M0), and absence of lymph node metastasis (N0) (**Figure 6B**).

**Figure 7:**
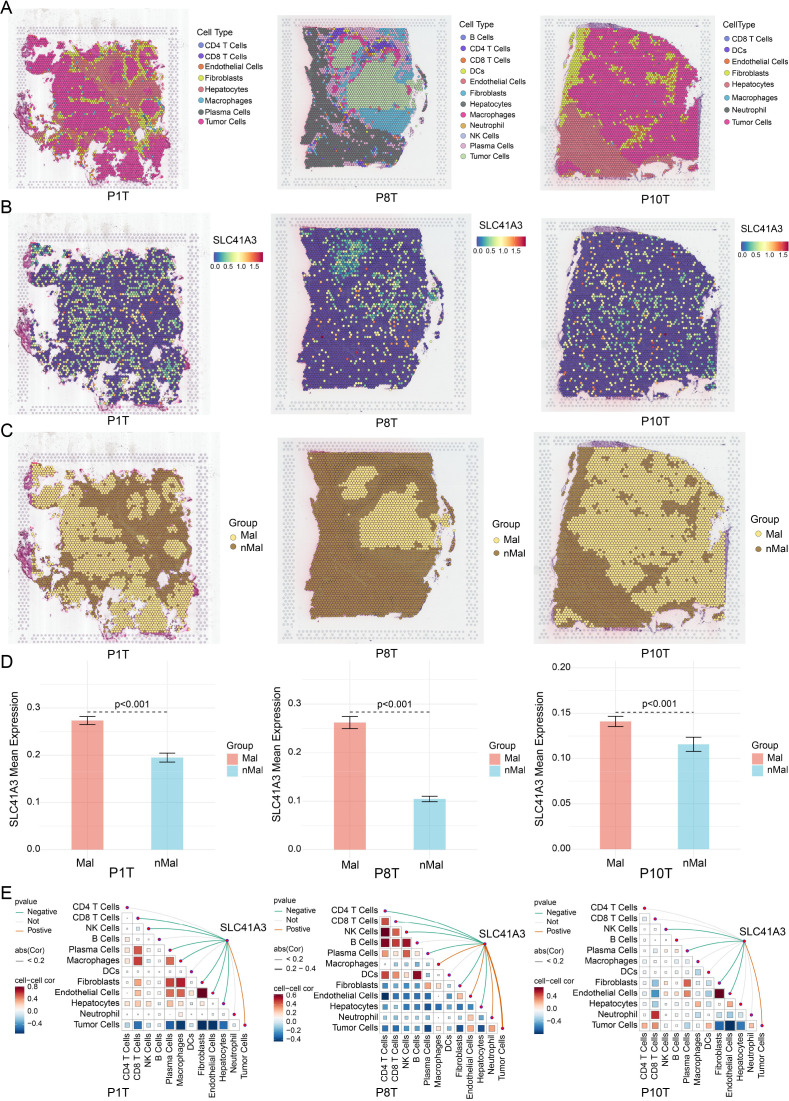
Spatial transcriptomic analysis of SLC41A3 expression in HCC patients. **(A)** Cellular distribution maps of tissue sections from three HCC patients. **(B)** Expression distribution of SLC41A3 across the sections. **(C)** Delineation of malignant and non-malignant cell populations within the tissue sections. **(D)** Bar plot depicting the expression levels of SLC41A3 within the malignant regions. **(E)** Spearman correlation analysis evaluating the relationship between the abundance of different cell types and SLC41A3 gene content in the spatial microenvironment.

To elucidate the spatial distribution of SLC41A3 expression within HCC tissues, we performed an analysis of spatial transcriptomic data derived from HCC tissue sections. Our findings demonstrated that SLC41A3 expression was markedly elevated in malignant regions relative to adjacent non-tumor areas, with its overall average expression also being significantly higher within tumor domains (**Figures 7A–D**). Subsequent Spearman correlation analysis reinforced these observations, revealing a positive association between SLC41A3 expression levels and the estimated proportion of tumor cells at individual spatial capture spots. Conversely, SLC41A3 expression showed negative correlations with the inferred abundance of several immune cell populations (**Figure 7E**). These results highlight the tumor-predominant expression signature of SLC41A3 and suggest its potential involvement in shaping the local TME.

### 3.6 Correlation of SLC41A3 expression with the immune microenvironment in HCC

To explore the association between SLC41A3 and the immunological context of HCC, patients within the TCGA-LIHC cohort were stratified into subgroups based on high versus low SLC41A3 expression. Comparative analysis revealed that the low SLC41A3 expression subgroup displayed a notable enrichment in the infiltration of several immune cell types, including cytotoxic cells, dendritic cells (DC), neutrophils, plasmacytoid dendritic cells (pDC), and NK CD56dim cells (**Supplementary Figure 6A**). This enhanced immune cell presence suggests a potential link to the more favorablesurvival outcomes observed in this patient group. Consistently, correlation analysis demonstrated aninverse relationship between SLC41A3 expression levels and the estimated enrichment scores of B cells, cytotoxic cells, DC, neutrophils, pDC, gamma delta T cells (Tgd), regulatory T cells (Treg), and T helper 17 (Th17) cells (**Supplementary Figure 6B**). Collectively, these analyses indicate that SLC41A3 may play a role in modulating immune cell recruitment and thereby influence the composition of the tumor microenvironment in HCC.

### 3.7 Potential biological roles of SLC41A3 in HCC

To elucidate the potential biological functions of SLC41A3, samples from the TCGA-LIHC cohortwere dichotomized into high- and low-expression groups based on the median expression level (top 50%vs. bottom 50%). Differential expression analysis between these groups (criteria: |log_2_FC| > 1, adjusted *P* value < 0.05) identified 926 upregulated and 324 downregulated mRNAs (**Supplementary Figure 7A**). KEGG pathway enrichment analysis revealed that these differentially expressed genes weresignificantly involved in processes such as Neuroactive ligand-receptor interaction, Bile secretion,and Retinol metabolism. Subsequent GO analysis further linked these genes to biological processes and molecular functions including regulation of hormone levels, digestion, collagen-containing extracellular matrix, “blood microparticle, and serine hydrolase activity (**Supplementary Figure 7B**). Moreover, GSEA demonstrated that high SLC41A3 expression was positively correlated withthe activation of signaling pathways including Pathways in cancer, Axon guidance, Wnt signalingpathway, and Gap junction (**Supplementary Figure 7C**).

### 3.8 SLC41A3 promotes malignant phenotypes *in vitro* of HCC

**Figure 8:**
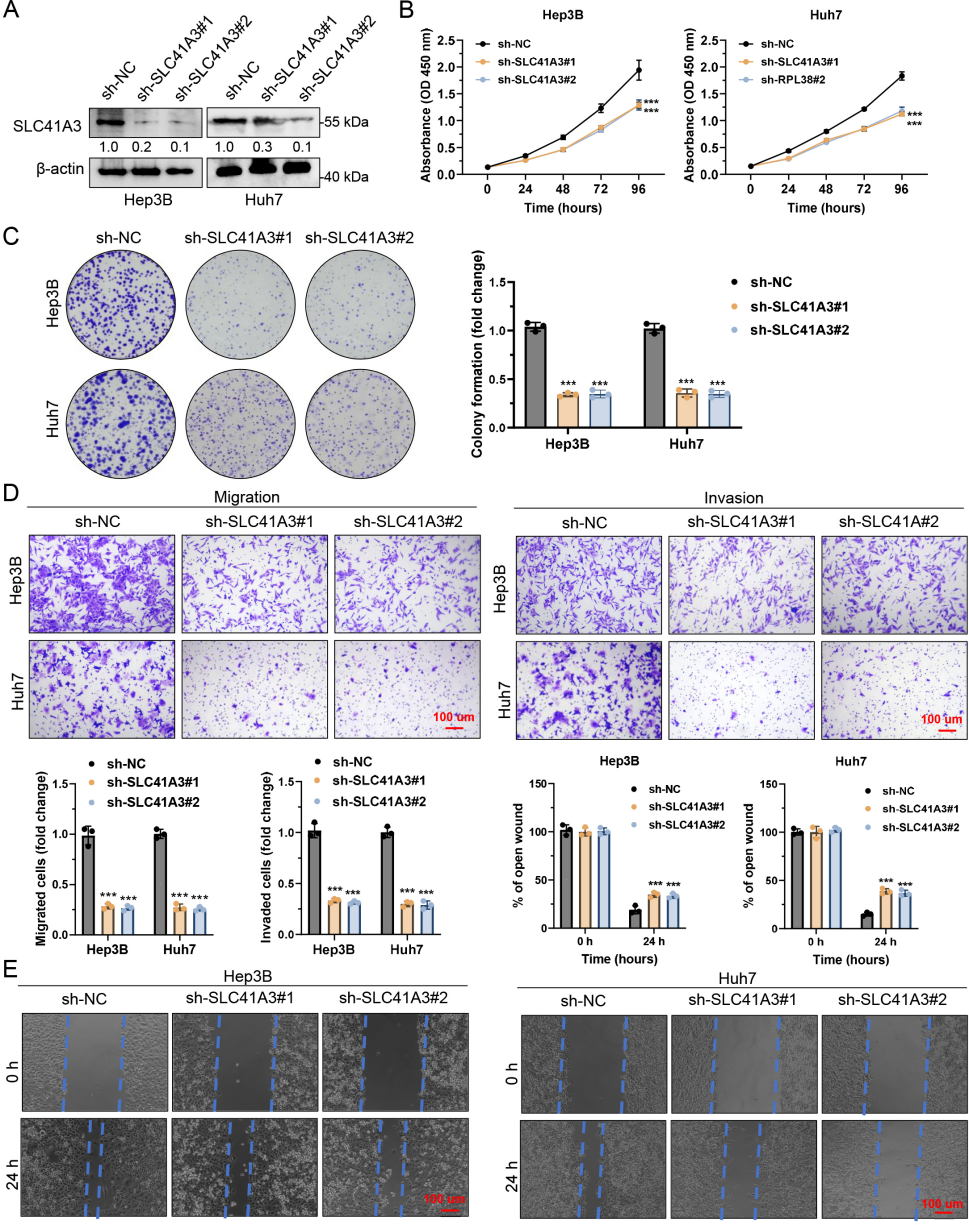
SLC41A3 promotes HCC progression. **(A)** Western blot analysis confirming the knockdown efficiency of SLC41A3 in Hep3B and HuH7 cells transfected with shRNA targeting SLC41A3. **(B)** CCK-8 assay evaluating cell proliferation at specified time points after SLC41A3 silencing in HCC cells. **(C)** Colony formation ability of Hep3B and HuH7 cells was inhibited after SLC41A3 knockdown. **(D)** Transwell migration and invasion assays indicated impaired migration and invasion capabilities after SLC41A3 downregulation in both cell lines. **(E)** Wound healing assay showed reduced cell motility after SLC41A3 knockdown. Scale bar, 100 μm. Data are presented as the mean ± SD of three independent experiments. *** *P* < 0.001.

Given the high expression of SLC41A3 in HCC, we performed stable knockdown of SLC41A3 in Hep3B and HuH7 cell lines using lentiviral shRNA to investigate its functional role. Effective depletion of SLC41A3 protein in both cell lines was confirmed by Western blotting (**Figure 8A**). Functionally, the CCK-8 assay revealed that SLC41A3 knockdown significantly impaired cell viability (**Figure 8B**). The colony formation assay further demonstrated a reduced clonogenic capacity in SLC41A3-depleted cells (**Figure 8C**). To investigate the contribution of SLC41A3 to cellular migratory and invasive properties, we performed Transwell migration and invasion assays alongside a scratch wound-healing assay. Depletion of SLC41A3 resulted in a substantial reduction in both the migratory and invasive capacities of the cells relative to controls (**Figure 8D**). Following SLC41A3 knockdown, the rate of wound closure was also observably impeded (**Figure 8E**). Taken together, these *in vitro* observations demonstrate that SLC41A3 silencing is capable of suppressing malignant phenotypes in HCC cells.

### 3.9 SLC41A3 knockdown suppresses HCC tumor growth *in vivo*

**Figure 9:**
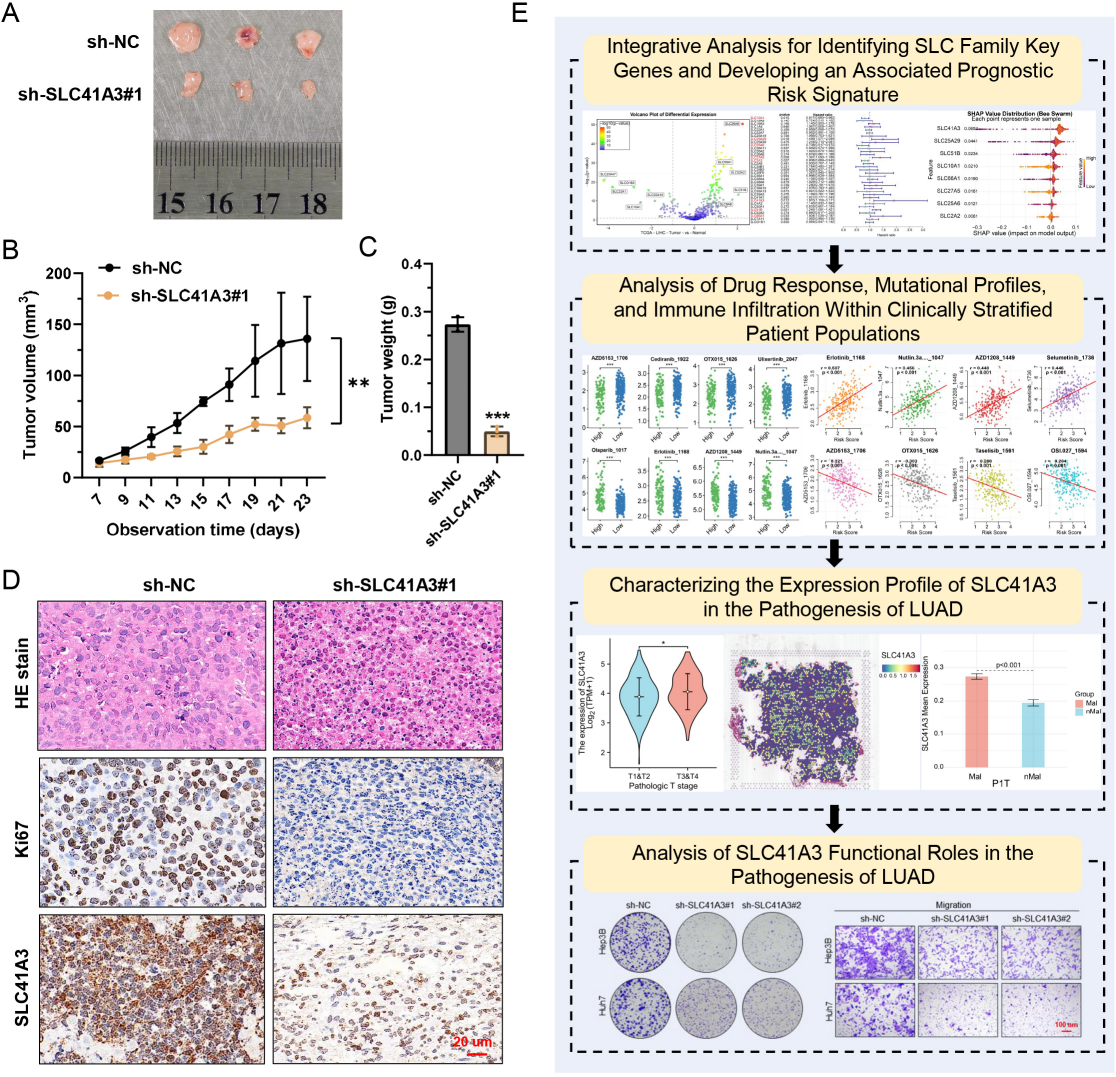
SLC41A3 knockdown inhibits HCC cell xenograft tumor growth. **(A)** Images of the harvested xenograft tumors on day 23. **(B)** Tumor growth curve. **(C)** The weight of xenograft tumors measured and plotted at 23 days post-inoculation. **(D)** IHC staining was used to examine changes in Ki-67 and SLC41A3 levels in the xenograft tumors. Representative images are shown. Scale bar, 20 μm. **(E)** A schematic summary depicting the main findings of this study. ** *P* < 0.01, *** *P* < 0.001.

The biological function of SLC41A3 was assessed *in vivo* using a subcutaneous xenograft model. Compared to mice inoculated with control (sh-NC) cells, those receiving SLC41A3-knockdown cells exhibited a significantly slower tumor growth rate over a 23-day observation period, as evidenced by attenuated tumor volume progression and a marked reduction in final tumor weight (**Figures 9A–C**). Subsequent immunohistochemical analysis confirmed that tumors derived from the SLC41A3-knockdown group showed downregulated expression of both SLC41A3 protein itself and the proliferation marker Ki67 (**Figure 9D**). A schematic diagram summarizing the key findings of this study is provided (**Figure 9E**). In summary, this study first identified pivotal SLC genes in HCC and constructed a robust prognostic signature based on them. Through integrated multi-omics approaches, we systematically validated the differential expression, clinical relevance, and immunomodulatory role of the core gene, SLC41A3. Finally, both *in vitro* and *in vivo* functional experiments established the tumor-promoting role of SLC41A3 in HCC pathogenesis.

## 4 Discussion

This study systematically reveals the critical role of SLC41A3 in HCC by integrating multi-omics data with experimental validation. We first identified SLC41A3 as a core gene significantly upregulated and independently associated with poor prognosis in HCC based on transcriptomic data from TCGA and GEO cohorts. Functional experiments demonstrated that knockdown of SLC41A3 effectively inhibited the proliferation and migration of HCC cells and induced cell cycle arrest. Experiments in immunodeficient mouse models further confirmed its tumor-promoting role *in vivo*. These results collectively establish SLC41A3 as an oncogenic driver in HCC.

The pro-tumorigenic function of SLC41A3 may be achieved through multi-layered mechanisms. First, its role in regulating cell cycle progression is consistent with phenotypic observations, as the G2/M phase arrest induced by knockdown directly explains the inhibition of proliferation. Second, as a magnesium ion transporter, SLC41A3 may exert broad effects on tumor biology by regulating intracellular magnesium ion homeostasis. Magnesium ions serve as cofactors for various key enzymes involved in energy metabolism, DNA synthesis, and cell signaling. Through an integrative bioinformatics approach, we discovered a prognostic model comprising eight SLC genes. In contrast to signatures derived from other gene families, this model’s emphasis on transporters that directly govern the flux of metabolites, ions, and therapeutic agents could provide a more proximate indicator of a tumor’s metabolic adaptations (16–18). SHAP interpretability analysis established SLC41A3 as the principal determinant within this model, thereby transforming its status from a sporadic prognostic correlate into an integral element of a composite molecular diagnostic framework. This advancement offers precise targets for the development of non-invasive clinical tools.

To contextualize our findings within existing knowledge, it is important to differentiate the role of SLC41A3 from other solute carriers implicated in cancer. Prior studies, including those on the DDR1-SLC1A5 axis (11), have established that certain SLC transporters can support tumor growth by fueling metabolic demands, such as glutamine uptake. Our study on SLC41A3 extends this paradigm by focusing on magnesium homeostasis—a less explored area in HCC—and by proposing a direct link to immune microenvironment remodeling. While SLC1A5 is primarily associated with nutrient supply for anabolic growth, our data position SLC41A3 as a potential immunomodulator via ion transport. This distinction underscores the novelty of our work: we not only identify a new prognostic SLC transporter but also propose a unique mechanism by which it may contribute to an immune-privileged niche, moving beyond the canonical metabolic support role attributed to many SLC family members.

Our proposed SLC family-based prognostic signature offers several advantages over existing models. First, its focus on SLC transporters, key regulators of the metabolic and immunologic landscape, provides a more mechanistic link to HCC biology. While other signatures may achieve comparable AUCs, our model is uniquely interpretable via SHAP analysis, which pinpointed SLC41A3 as the core biomarker. The lack of validation in an independent dataset, such as from the ICGC or other public repositories, is a limitation of the current study. Future work is needed to externally validate the model to strengthen its clinical relevance.

The genomic alterations analysis demonstrated distinct mutational profiles between the risk groups: mutations in *TP53* were predominantly found within the high-risk cohort, whereas *CTNNB1* alterations showed a higher frequency in the low-risk group (19, 20). This observation offers a critical mechanistic foundation, given that TP53 loss-of-function is associated with heightened genomic instability and more aggressive tumor phenotypes. Here, we hypothesize that inactivation of the TP53 signaling pathway might indirectly reprogram tumor metabolism through the modulation of select SLC family transporters, culminating in reduced therapeutic sensitivity—a notion supported by analogous research showing DDR1 stabilizes SLC1A5 to drive HCC progression (21). Critically, this *TP53*-mutant landscape, particularly its enrichment in the high-risk group, may also directly contribute to shaping an immunosuppressive tumor microenvironment. *TP53* loss-of-function is known to promote the secretion of immunosuppressive cytokines and chemokines, thereby fostering an immune-cold phenotype that facilitates tumor immune evasion.

TIME characterization further demonstrated that the high-risk cohort displayed features consistent with an immunosuppressive landscape. This was evidenced by a relative enrichment of regulatory T cells (Tregs) and T helper 2 (Th2) cells, coupled with increased expression of chemokines such as CCL2 and CCL5 (22–25). This analysis was performed using the CIBERSORT deconvolution algorithm. To assess the robustness of this finding, we also applied an alternative method (xCell), which yielded a consistent pattern of immune cell abundance differences between the risk groups, particularly supporting the observed enrichment of immunosuppressive cell types in the high-risk cohort. Such an “immune-cold” phenotype corresponds to molecular subtypes previously linked to unfavorable outcomes by other biomarker studies (26, 27). Furthermore, based on the established knowledge that extracellular magnesium levels can modulate T cell function and immune synapse stability (28–30), we hypothesize that SLC41A3, by potentially altering local magnesium availability, might contribute to the observed immunosuppressive TME signature associated with its high expression. This signature includes a positive correlation with regulatory T cell (Treg) infiltration and a negative correlation with cytotoxic T cell infiltration. However, this remains a speculative model, and direct experimental evidence causally linking SLC41A3 activity to magnesium-dependent immune cell suppression in the HCC TME is currently lacking. Specifically, we did not measure or cite actual magnesium concentrations in tumor tissues, which represents a critical gap in the current evidence chain. This limitation, and the need for future metabolomic studies or direct ion quantification, represents an important avenue for future investigation.

Our analysis further indicated that the SLC gene signature we developed was associated with increased sensitivity to Olaparib, a PARP inhibitor, hinting at a potential therapeutic implication for this HCC subgroup. It is important to interpret this association with nuance. The predicted sensitivity is likely an indirect association, primarily reflecting the well-established link between TP53 mutations and response to PARP inhibitors, rather than a direct, SLC41A3-specific mechanism. Our signature incorporates SLC family genes, and its correlation with TP53 mutational status is probably the main driver of the drug response prediction. Therefore, while the signature holds predictive value, it should not be construed as evidence that SLC41A3 per se directly sensitizes cells to Olaparib. Future work employing isogenic cell models with modulated SLC41A3 expression is needed to dissect its specific role, independent of the TP53 background. Concurrently, we cannot rule out that differential expression of SLC transporters might influence intracellular drug distribution or pharmacokinetics—a mechanism documented for other family members such as SLC41A1 (31, 32). By integrating both genomic alteration data (*TP53* status) and transcriptional profiles (SLC signature), our model may capture these complementary layers of biological information, thereby offering a more robust framework for predicting therapeutic vulnerability.

Pan-cancer investigation demonstrated that SLC41A3 exhibits widespread transcriptional upregulation across multiple malignancies. This pattern was accompanied by frequent amplification of its gene copy number and alterations in DNA methylation patterns, suggesting its promise as a pan-cancer biomarker, paralleling the behavior of established pan-cancer markers such as RAB42 (33, 34). It is important to note that this pan-cancer analysis was limited to profiling SLC41A3 expression and associated genomic/epigenomic features. A logical and valuable next step would be to investigate whether the association between high SLC41A3 expression and an immunosuppressive microenvironment, as observed in HCC, is consistent across other cancer types. For instance, comparing its role in traditionally immune-’hot’ tumors (melanoma or certain subtypes of lung cancer) versus immune-’cold’ tumors (like HCC or pancreatic ductal adenocarcinoma) could reveal whether SLC41A3’s immunomodulatory function is cancer-type specific or a more general phenomenon. Such comparative analyses remain an important avenue for future research.

From a clinical translation perspective, the expression level of SLC41A3 provides a new potential biomarker for HCC prognosis assessment. The prognostic nomogram we constructed, which includes SLC41A3, demonstrated stable predictive performance in both the training and external validation sets. Importantly, our risk model exhibits incremental prognostic value beyond conventional clinical staging systems and liver function scores. While the Barcelona Clinic Liver Cancer (BCLC) staging and tumor-node-metastasis (TNM) classification are standard tools for HCC prognosis, they primarily rely on anatomical tumor burden and liver function, and may not fully capture the underlying biological heterogeneity of the disease. Similarly, the Albumin-Bilirubin (ALBI) and Child-Pugh scores assess hepatic reserve but do not reflect tumor-intrinsic molecular characteristics. Our SLC gene-based signature, derived from tumor transcriptomic profiles, provides orthogonal information on the tumor’s metabolic and immunologic state. For example, even among patients with the same BCLC stage, our model could further stratify those at higher risk of poor outcomes, thereby guiding individualized treatment decisions. Nonetheless, we acknowledge that the current analysis is based on retrospective data and the true clinical utility of integrating our model into routine practice requires prospective validation in cohorts with standardized staging and treatment protocols. This limitation restricts the generalizability of our prognostic model to broader, real-world patient populations. Future studies should prioritize the collection and analysis of independent clinical cohorts, particularly from diverse ethnicities and treatment settings, to rigorously assess the model’s performance and ensure its clinical applicability. Compared to some published prognostic signatures (35, 36), our model not only incorporates genes with clear biological functions but also ensures the reliability of core genes through cross-validation across multiple databases and provides a quantifiable tool for clinical use, which are its advantages.

The spatial transcriptomics analysis in our study provides a valuable layer of evidence, revealing a distinctive pattern of “mutual exclusion” between regions with high SLC41A3 expression and those enriched for immune cell transcripts. This observation is intriguing and aligns with the functional role we propose for SLC41A3 in shaping an immunosuppressive microenvironment. However, we recognize that this spatial pattern must be interpreted with caution. Several technical and biological confounders could contribute to this observation. For instance, areas of tissue necrosis, which are common in rapidly growing tumors, may simultaneously exhibit low expression of both tumor-specific genes and immune cell markers due to cell death, creating a false impression of co-occurrence rather than exclusion. Similarly, regional variations in overall cell density can skew transcript counts; a region with high tumor cell density might artificially show lower relative abundance of immune transcripts. Furthermore, the inherent resolution limits of spatial transcriptomics in our current analysis may not fully capture the fine-scale cellular architecture of the tumor microenvironment. Therefore, while our findings are supportive, the “mutual exclusion” pattern should be viewed as a hypothesis-generating spatial correlation, not a definitive proof of a causal immune-exclusion mechanism. Future studies employing higher-resolution spatial profiling or multiplexed protein-based imaging are needed to validate these findings. From a translational perspective, our findings also suggest that targeting SLC41A3, potentially with specific inhibitors, in combination with immune checkpoint blockade could represent a promising therapeutic strategy for HCC patients with high SLC41A3 expression. Future preclinical studies using immunocompetent models are warranted to evaluate the efficacy of such combination regimens and to determine whether SLC41A3 inhibition can enhance antitumor immunity by reversing the immunosuppressive microenvironment.

This study is subject to several limitations. First, concerning the robustness of the prognostic model, we acknowledge the inherent risk of overfitting associated with machine learning-based feature selection, particularly given the relatively limited sample size coupled with the high-dimensional feature space. Although we employed Lasso regression with internal cross-validation to mitigate this risk, the potential for capitalizing on stochastic correlations remains, and the model’s generalizability to truly independent, heterogeneous populations warrant further rigorous evaluation. Second, our integrative analysis amalgamated data from multiple public cohorts (TCGA, GEO). Despite implementing standard normalization pipelines, latent batch effects arising from disparities in sequencing platforms, sample processing protocols, and institution-specific practices may confound the biological signals. While we applied batch correction algorithms (ComBat), residual technical variation cannot be entirely excluded, which may influence the stability of the identified features. Third, our survival analyses, including the construction of Cox models and Kaplan-Meier curves, employed traditional methods that treat non-cancer-related deaths (from liver failure) as censored events. In HCC, where such competing risks are frequent, this approach may overestimate the cumulative incidence of cancer-specific death. Future studies with detailed cause-of-death information should employ competing risk models (the Fine-Gray model) to validate and refine our prognostic findings. Fourth, although we performed external validation using an independent dataset (GSE244826), this cohort does not represent a prospective, multi-center trial with standardized interventions. The absence of validation in a rigorously controlled, real-world clinical setting limits the immediate translational applicability of our model. Fifth, while we corroborated elevated SLC41A3 protein expression using public immunohistochemistry data (HPA), independent validation with a dedicated clinical cohort is necessary. Future investigations employing such models could explore the therapeutic potential of targeting SLC41A3, particularly in combination with immunotherapies (immune checkpoint inhibitors), to evaluate synergistic effects on tumor growth and immune landscape remodeling. Seventh, the hypothesized mechanism by which SLC41A3 shapes an immune-privileged niche through modulation of extracellular magnesium, while grounded in established knowledge, remains speculative and requires direct experimental validation in immunocompetent models. Finally, as a transporter, the specific downstream effector molecules and comprehensive signaling networks regulated by SLC41A3 merit further elucidation.

In summary, this study implemented a novel analytical framework that synergistically combined machine learning with multi-omics data to establish a reliable prognostic signature derived from the SLC transporter family. Our work provides the first comprehensive evidence, substantiated by spatial transcriptomics and functional assays, for the central role of SLC41A3 as a driver of oncogenesis and a regulator of the immune milieu. These results yield a novel prognostic instrument and identify a potential therapeutic target in HCC. They advance the mechanistic comprehension of hepatocarcinogenesis and establish a conceptual foundation for innovative therapeutic combinations aimed at the SLC41A3-associated immunometabolic network. Subsequent investigations should aim to elucidate the specific immunomodulatory pathways governed by SLC41A3 using immunocompetent model systems and to pursue the development of targeted inhibitors.

## 5 Conclusion

This integrative study establishes SLC41A3 as a pivotal oncogene and a robust prognostic biomarker in HCC. Elevated SLC41A3 expression correlates with aggressive clinicopathological features and predicts unfavorable patient survival. We provide direct experimental validation demonstrating that SLC41A3 drives HCC cell proliferation and tumorigenesis. Moreover, we unveil a novel link between SLC41A3 and the shaping of an immunosuppressive tumor microenvironment. Our findings not only advance the understanding of SLC transporters in liver cancer pathogenesis but also nominate SLC41A3 as a valuable tool for prognostic stratification. While SLC41A3 represents a promising therapeutic target, we acknowledge the substantial druggability challenges inherent to magnesium transporters, including structural complexity and selectivity issues. Therefore, future investigations should prioritize rigorous assessments of target feasibility—exploring alternative modalities such as antibody-drug conjugates, protein degradation strategies, or genetic approaches—over simply reiterating therapeutic potential. Specifically, efforts should focus on elucidating the precise mechanisms by which SLC41A3 orchestrates metabolic reprogramming and immune evasion in HCC, thereby providing a more rational foundation for future translational development.

## Data Availability

The original contributions presented in the study are included in the article/**Supplementary Material**. Further inquiries can be directed to the corresponding authors.
