# Supplementary figures and images for: Comprehensive characterization of SLC41A3 identifies it as an immune-related prognostic biomarker and therapeutic target in hepatocellular carcinoma
Source: Front Immunol. 2026 Jun 3;17:1861310. doi: 10.3389/fimmu.2026.1861310 (PMC13272485; doi:10.3389/fimmu.2026.1861310)

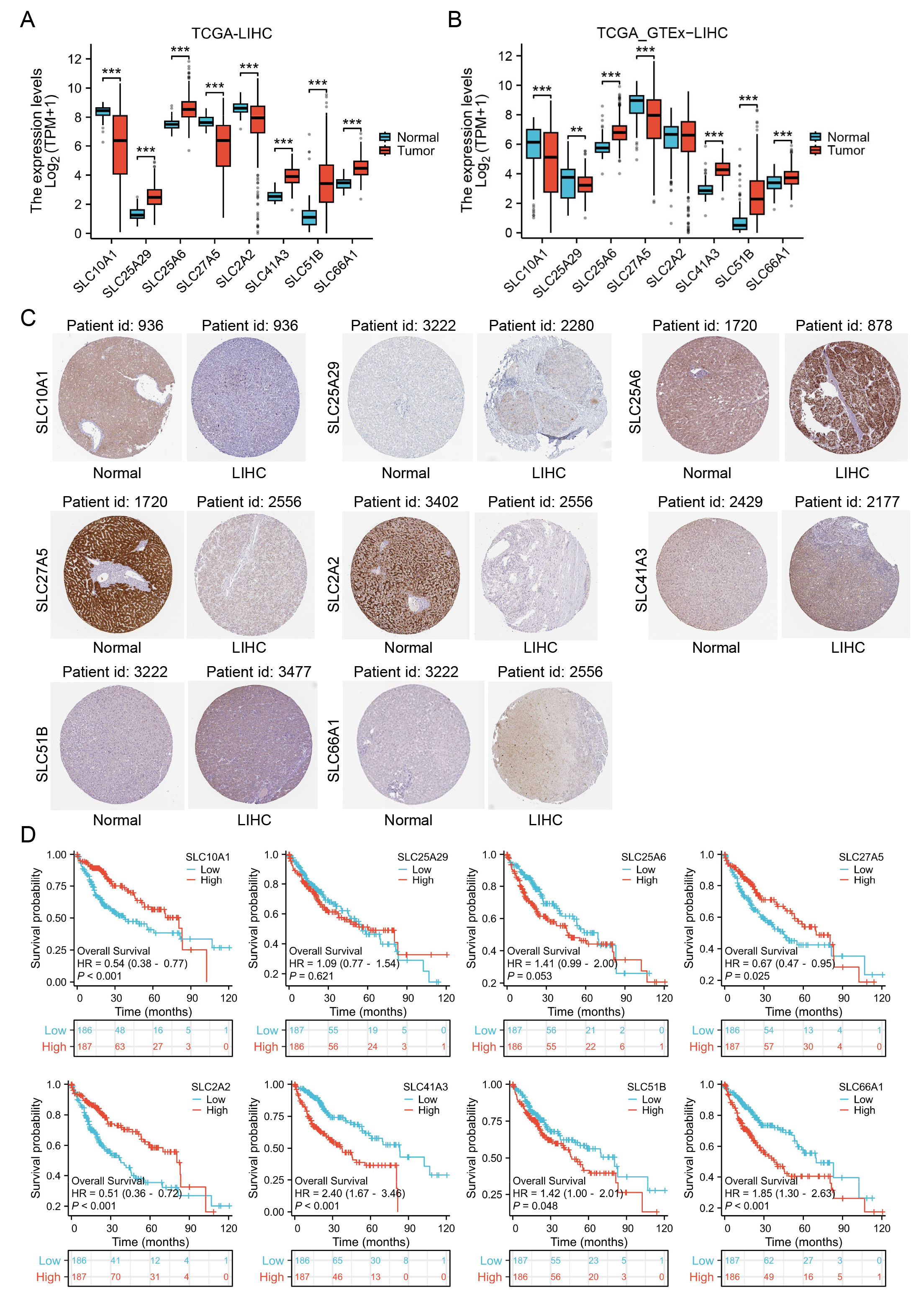

Supplement: Supplementary file 1 [file Image1.jpeg]

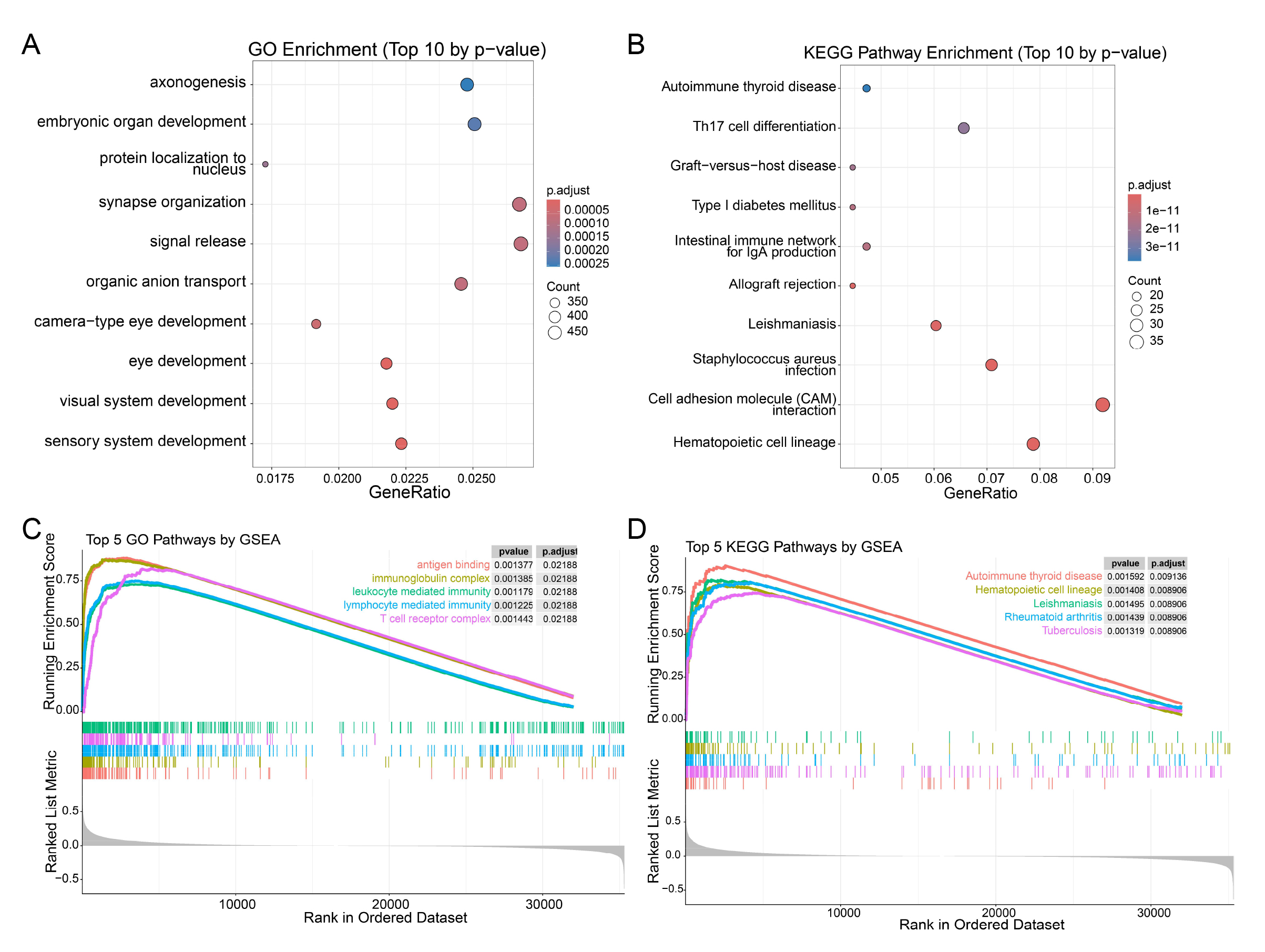

Supplement: Supplementary file 2 [file Image2.jpeg]

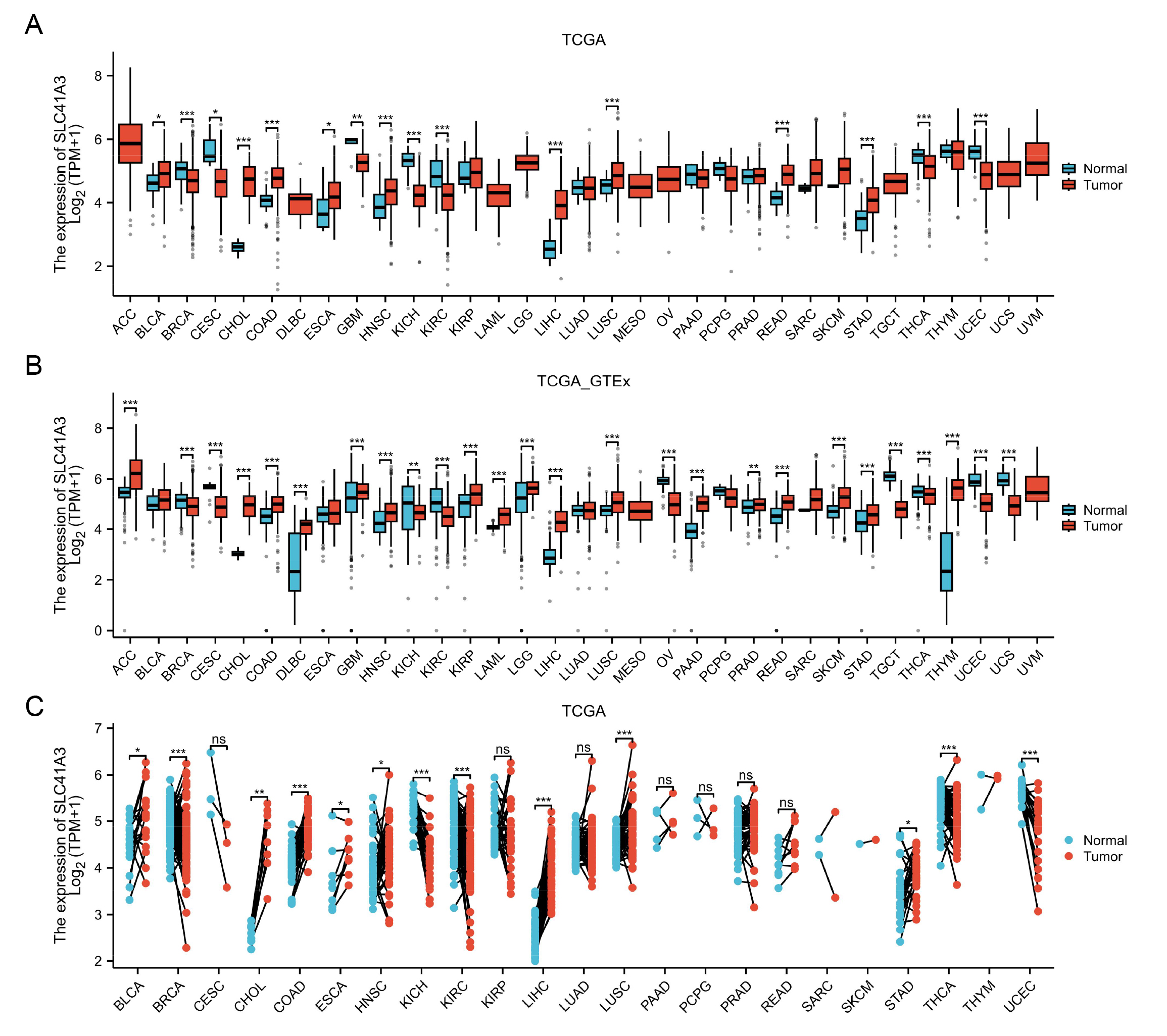

Supplement: Supplementary file 3 [file Image3.jpeg]

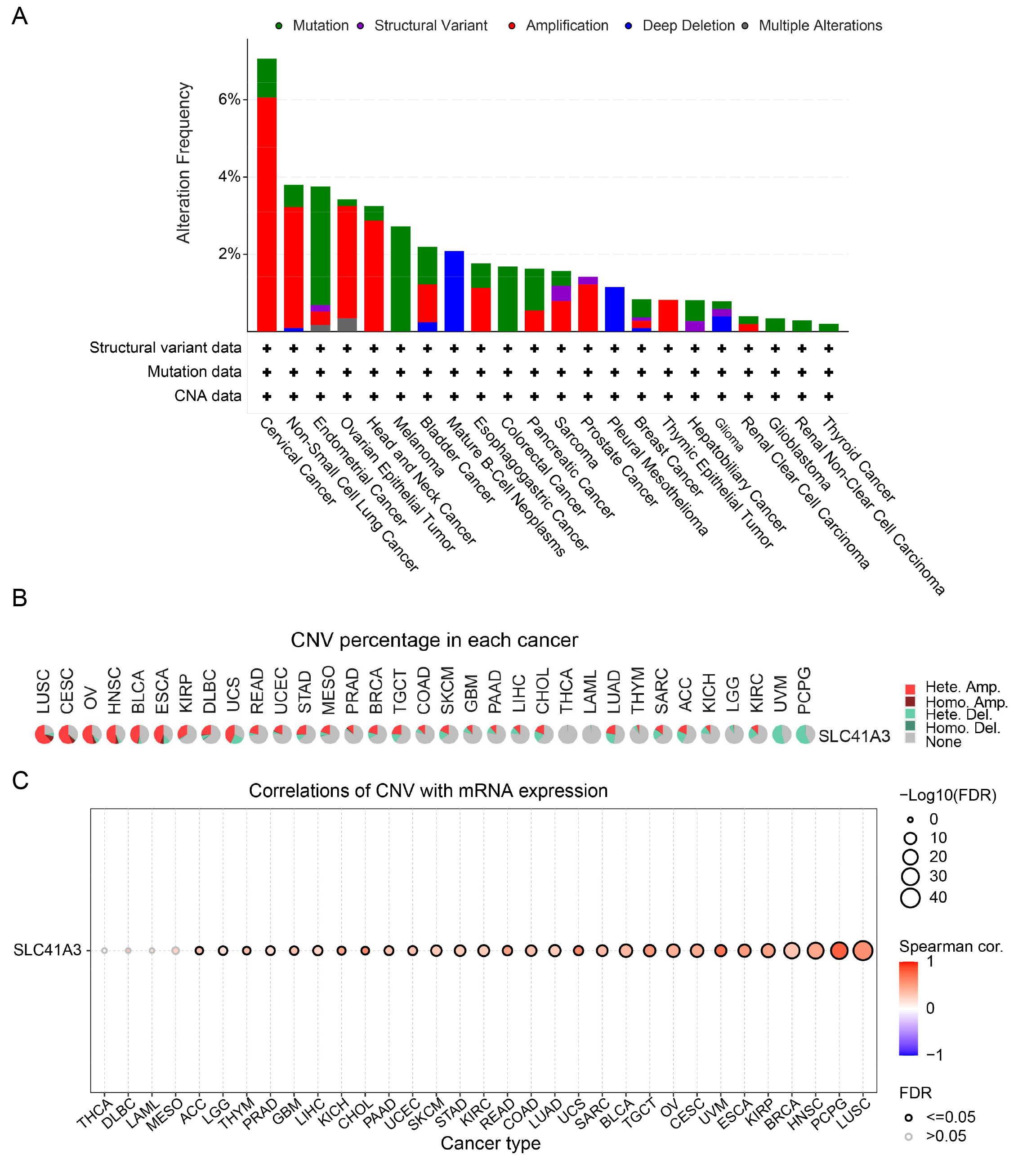

Supplement: Supplementary file 4 [file Image4.jpeg]

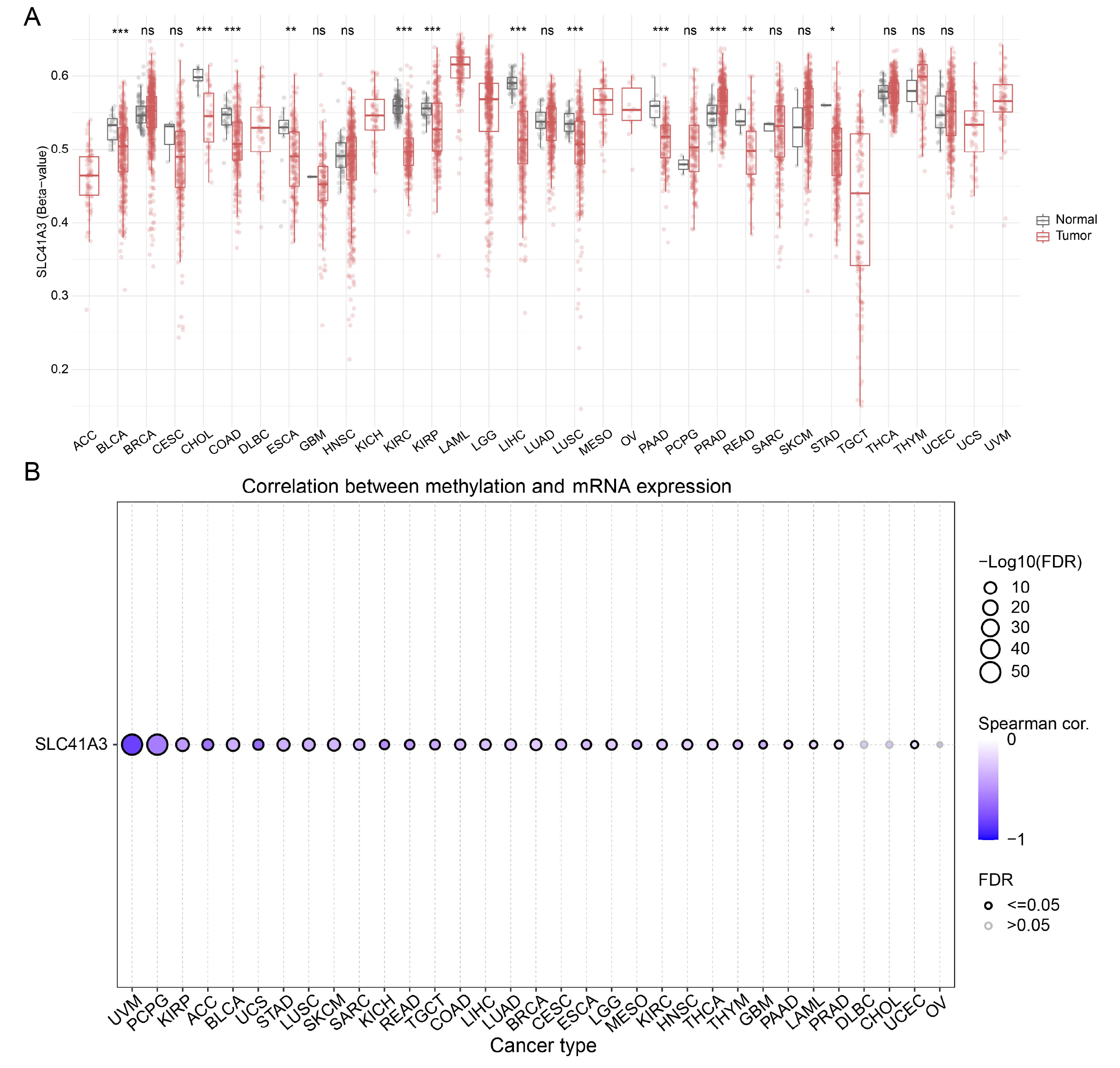

Supplement: Supplementary file 5 [file Image5.jpeg]

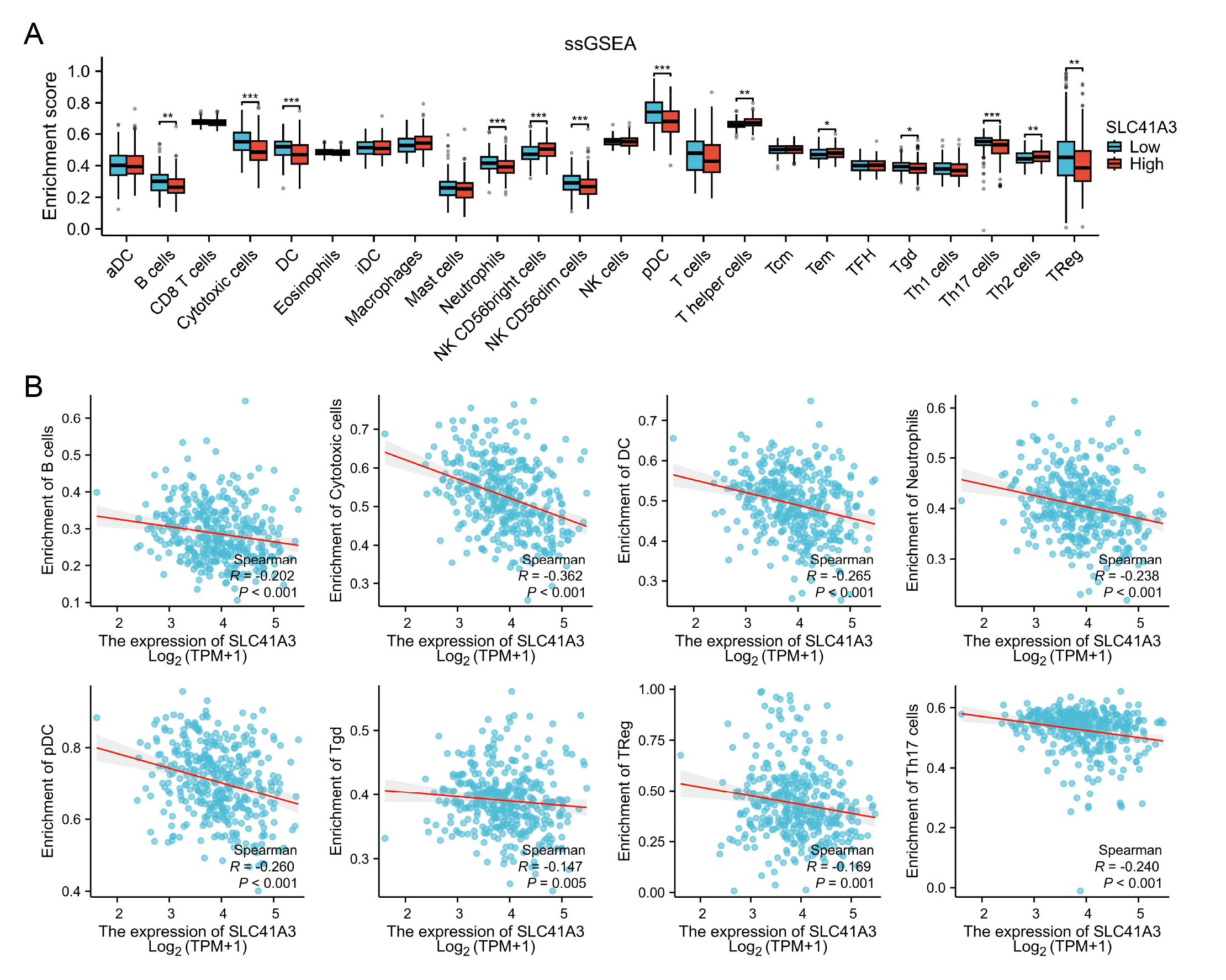

Supplement: Supplementary file 6 [file Image6.jpeg]

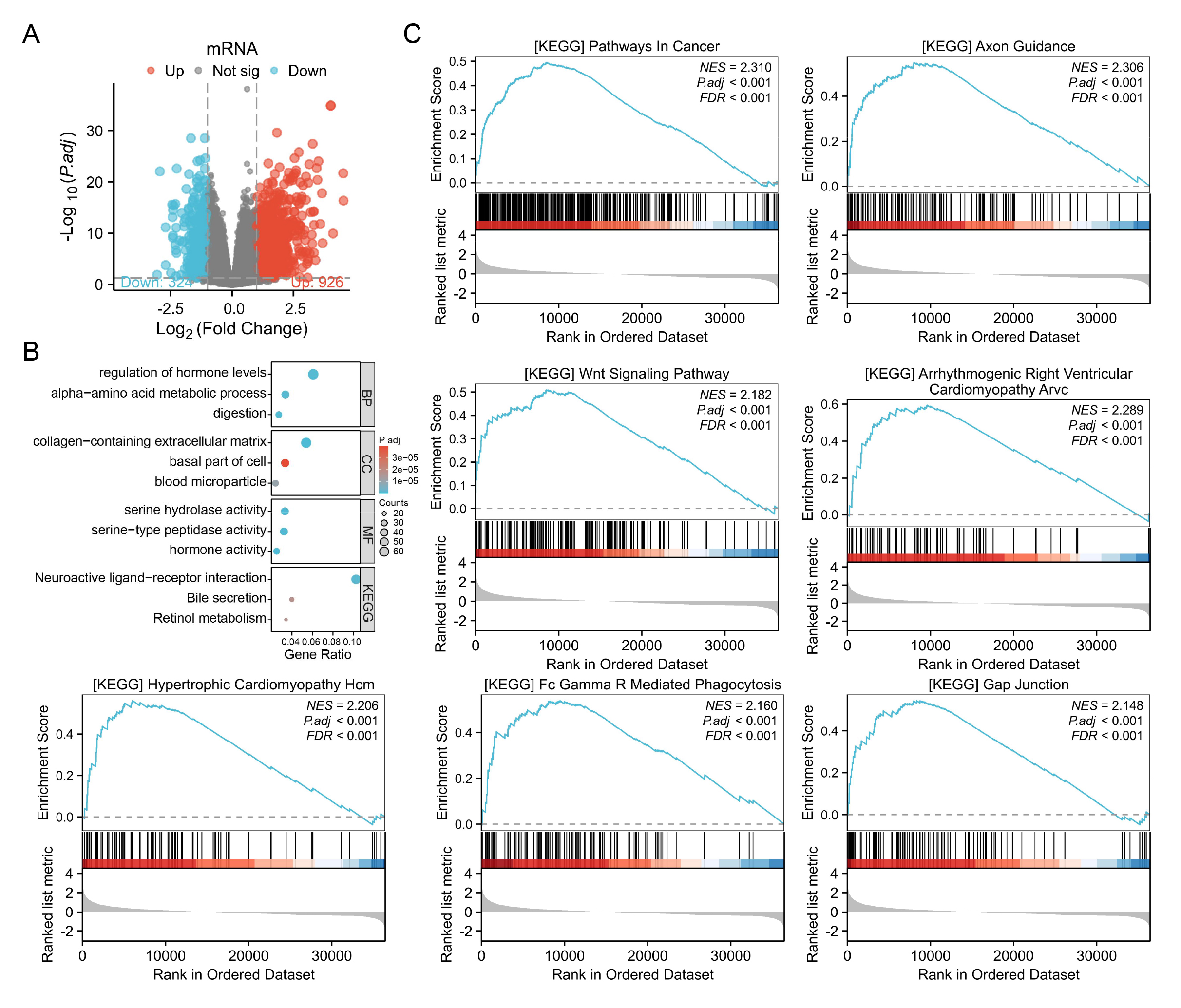

Supplement: Supplementary file 7 [file Image7.jpeg]

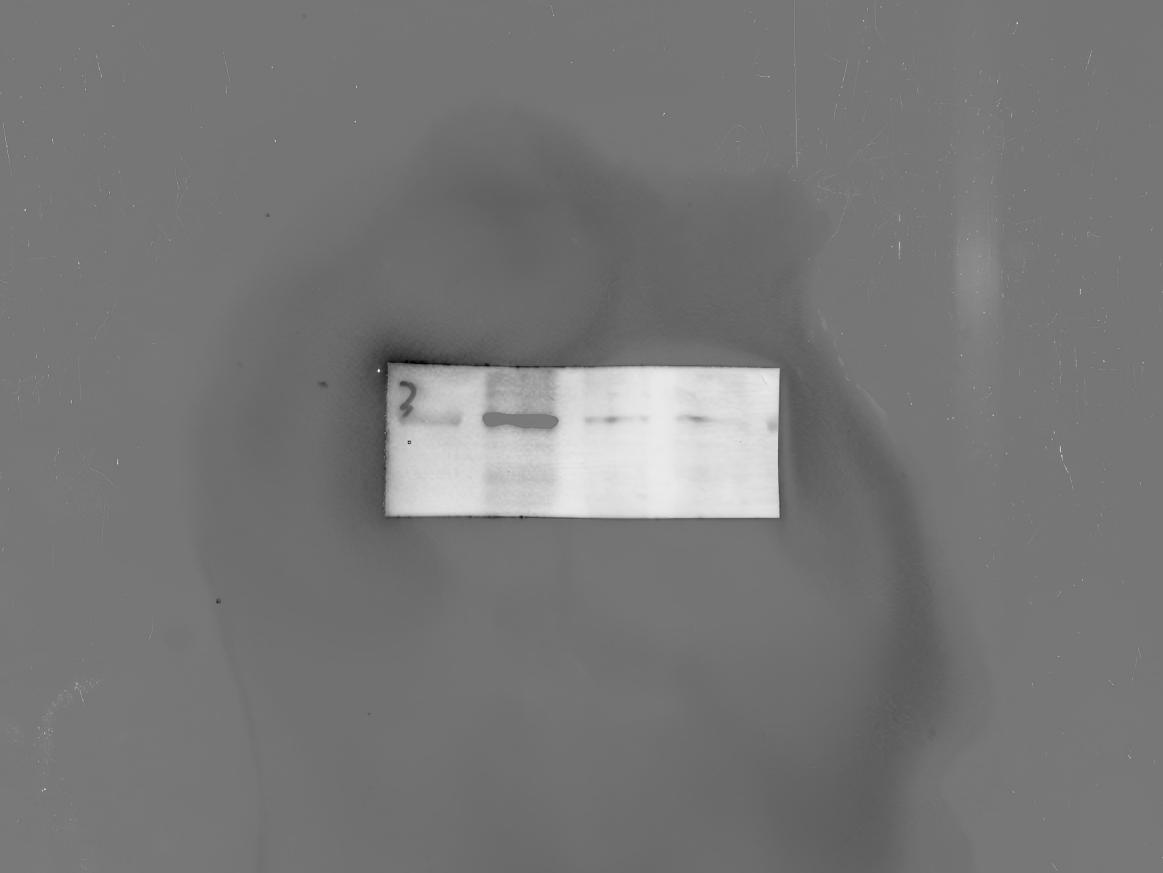

Supplement: Supplementary file 8 [file DataSheet1.zip › WB/Hep3B-SCL41A3.jpg]

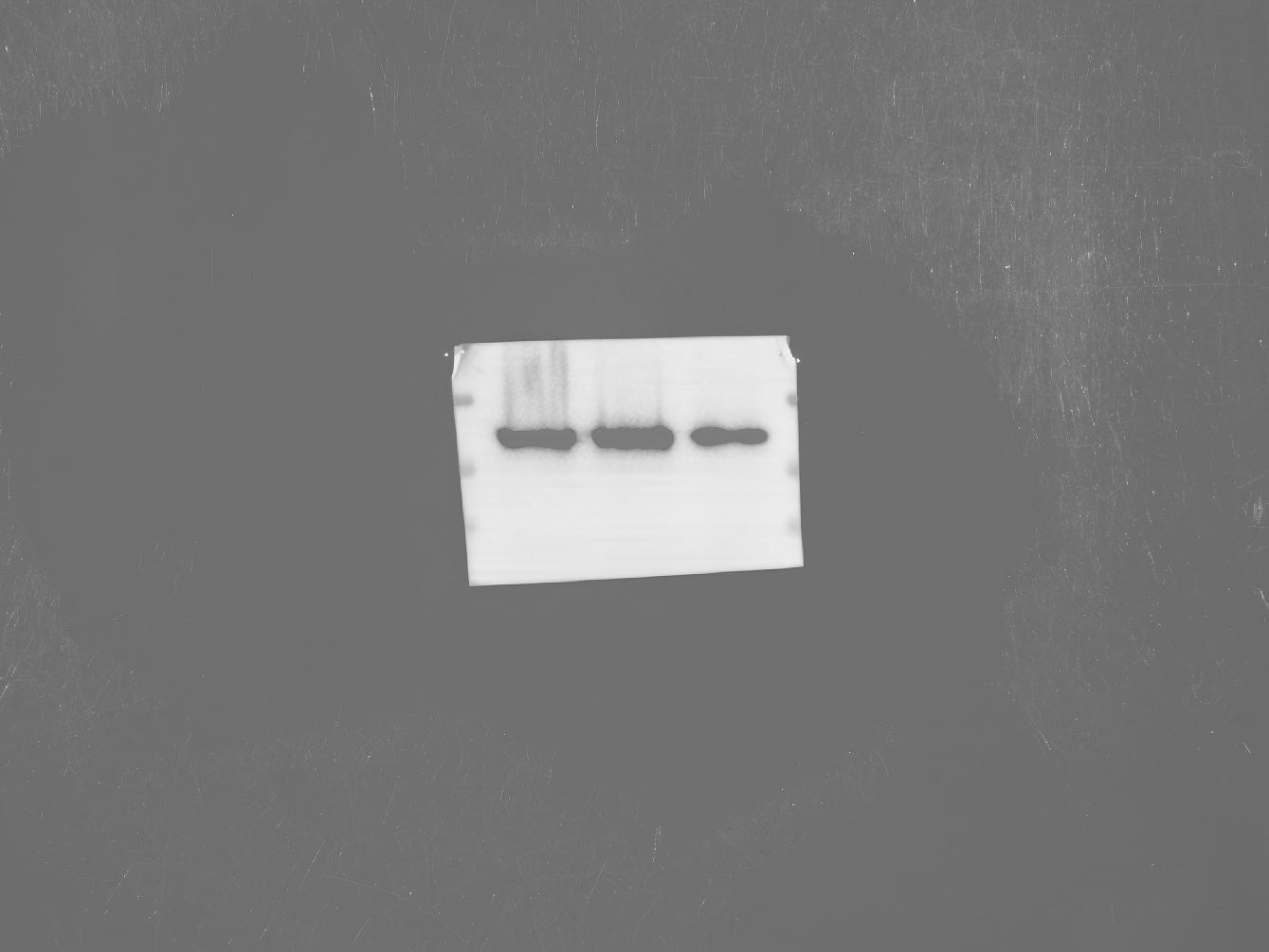

Supplement: Supplementary file 8 [file DataSheet1.zip › WB/Hep3B-b-actin.jpg]

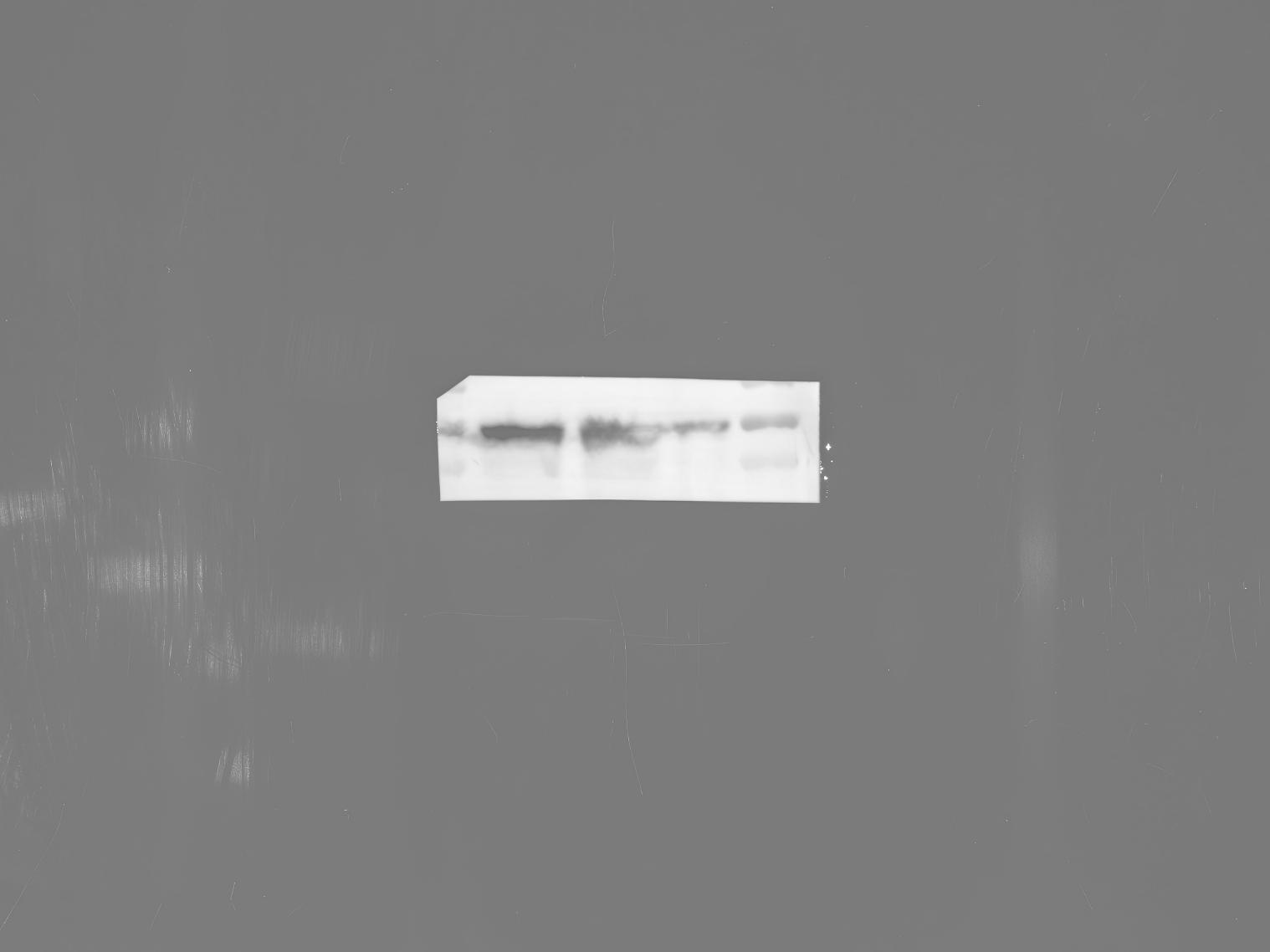

Supplement: Supplementary file 8 [file DataSheet1.zip › WB/Huh7-SLC41A3.jpg]

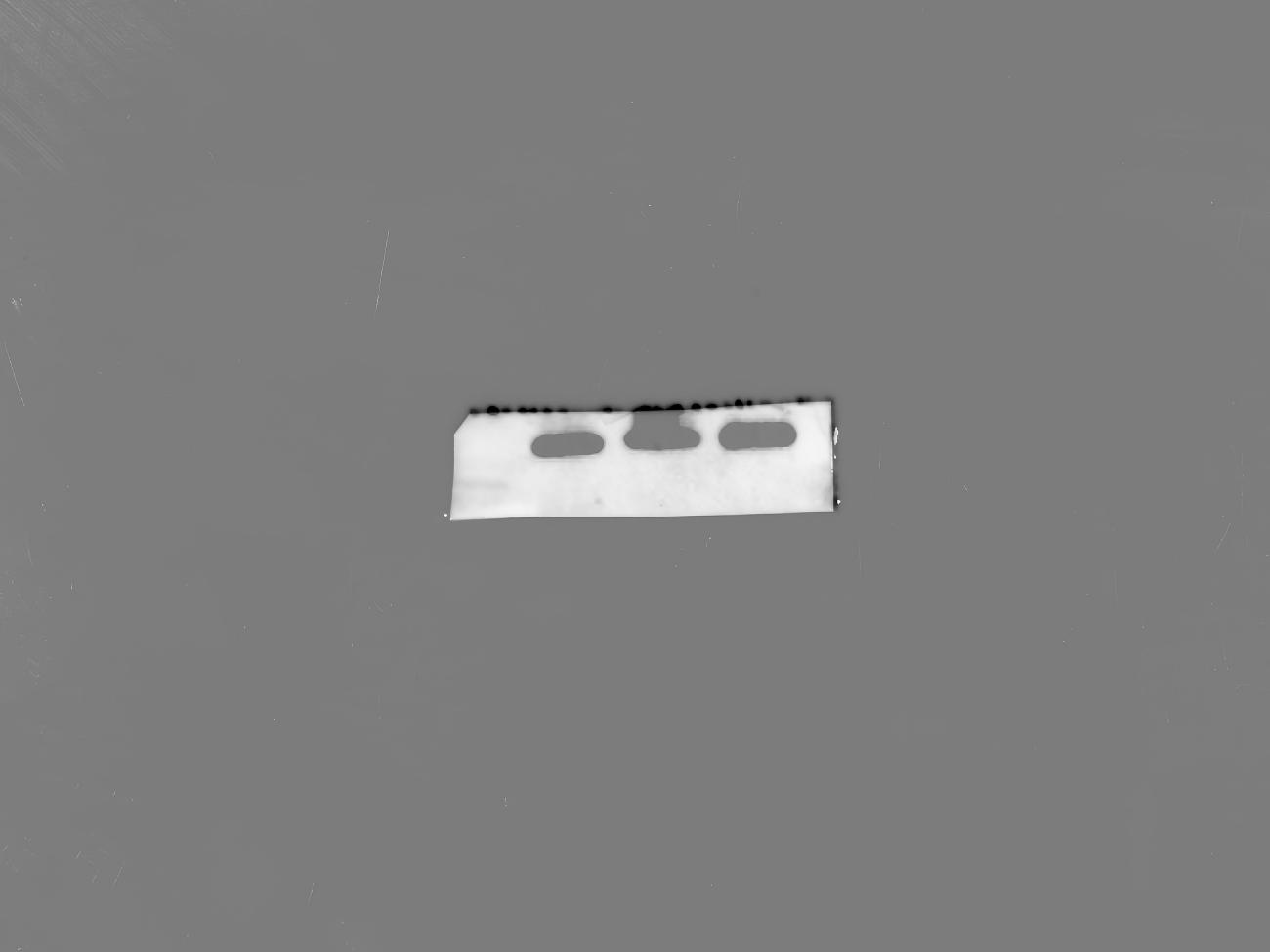

Supplement: Supplementary file 8 [file DataSheet1.zip › WB/Huh7-b-actin.jpg]
