# Supplementary material for: Comprehensive characterization of SLC41A3 identifies it as an immune-related prognostic biomarker and therapeutic target in hepatocellular carcinoma
Source: Front Immunol. 2026 Jun 3;17:1861310. doi: 10.3389/fimmu.2026.1861310 (PMC13272485; doi:10.3389/fimmu.2026.1861310)
Supplement: Supplementary file 9 [file DataSheet2.docx]

**Comprehensive Characterization of SLC41A3 Identifies It as an Immune-Related Prognostic Biomarker and Therapeutic Target in Hepatocellular Carcinoma**


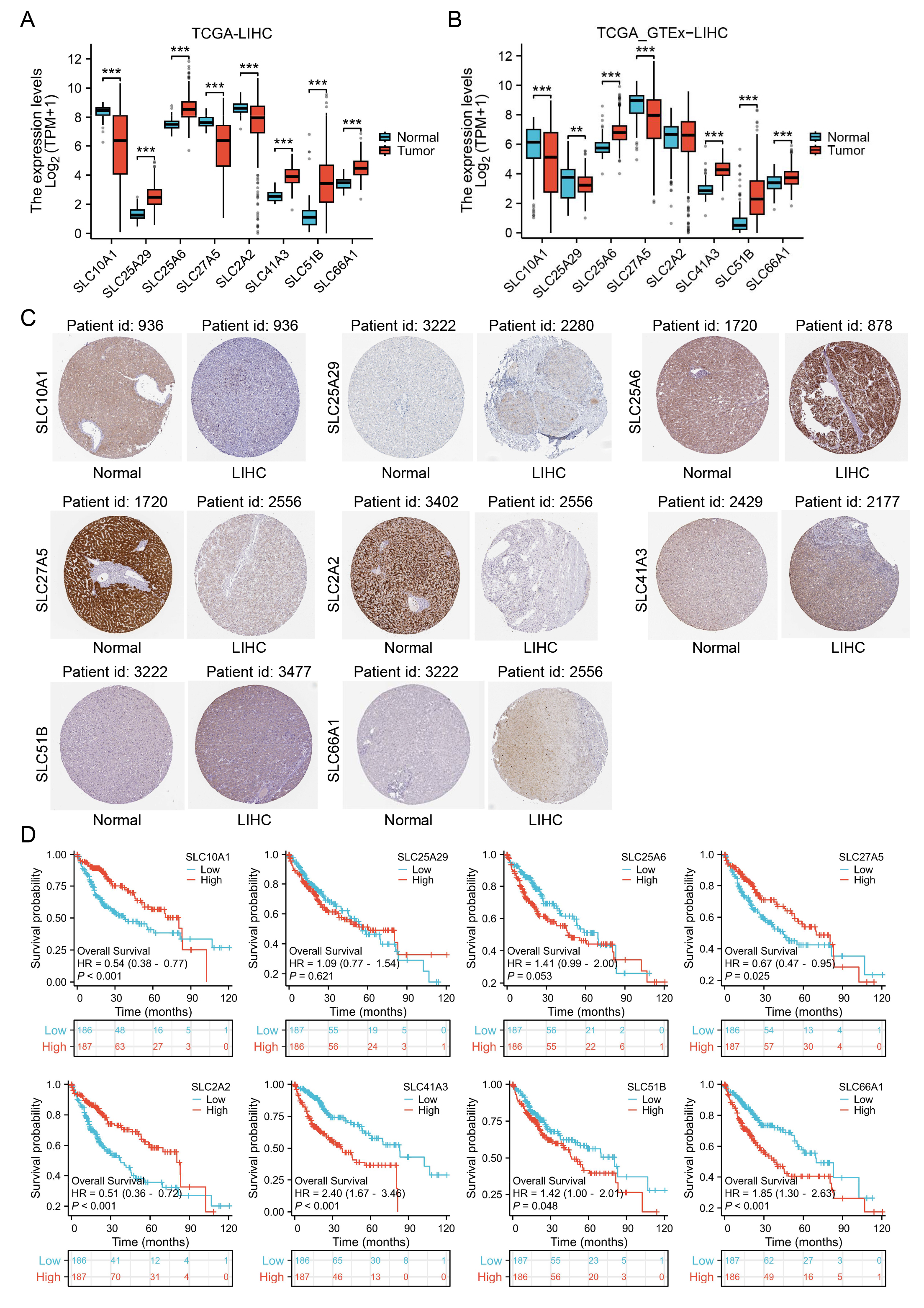


**Supplemental Figure 1. Expression and prognostic analysis of key genes in hepatocellular carcinoma.**

(A-B) Expression analysis of the 8 candidate genes in the TCGA-LIHC cohort (A) and the combined TCGA-LIHC and GTEx database (B). (C) Immunohistochemical validation of protein expression patterns in HCC using the Human Protein Atlas. (D) Kaplan-Meier survival analysis evaluating the prognostic significance of the 8 candidate genes in LIHC. *** *P* <0.001.


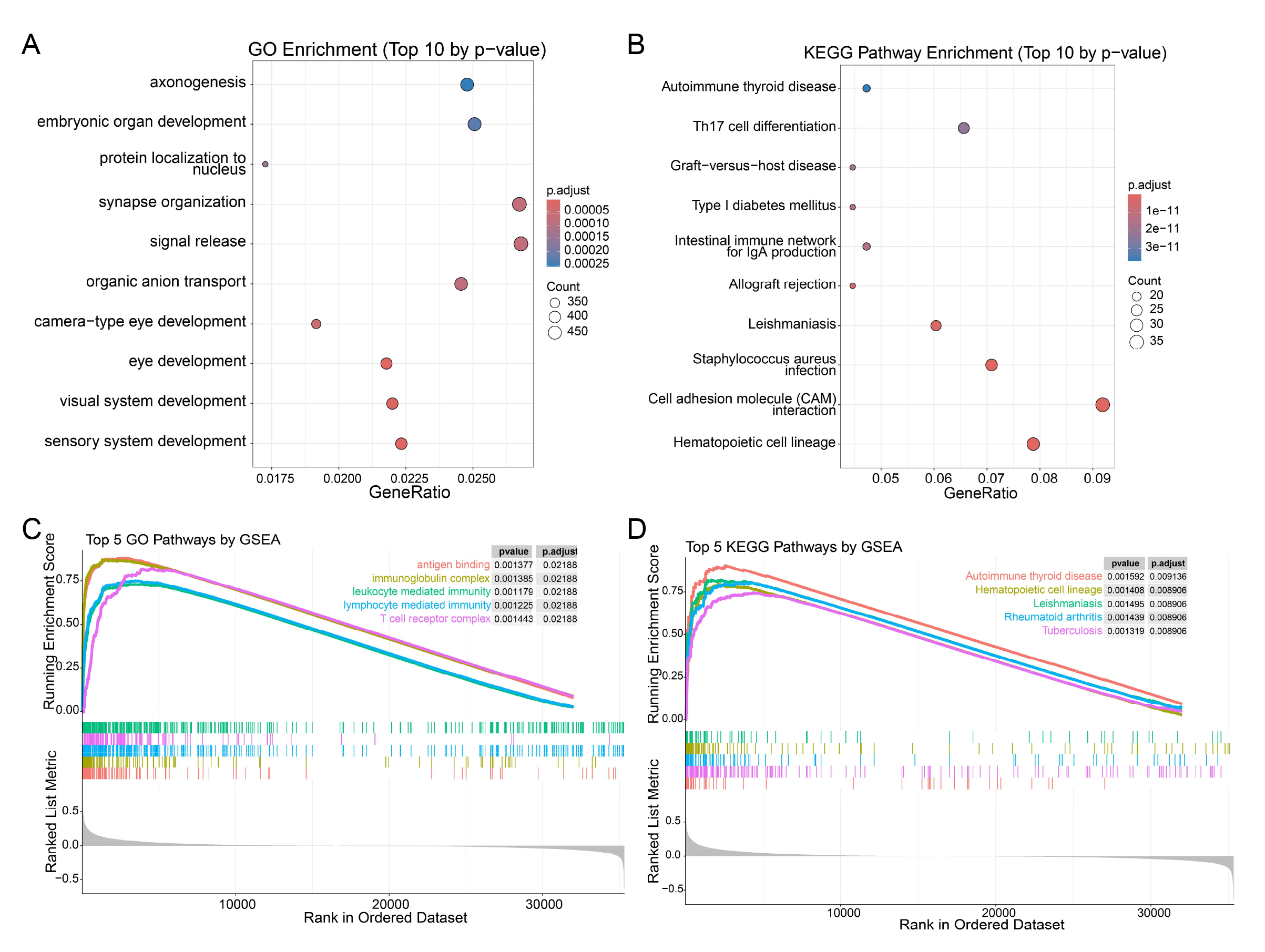


**Supplemental Figure 2. Functional enrichment analysis of differentially expressed genes between high- and low-risk groups.**

(A-B) GO and KEGG pathway enrichment analysis of differentially expressed genes between the high- and low-risk groups. (C-D) GSEA enrichment analysis of the differentially expressed genes between the high- and low-risk groups.


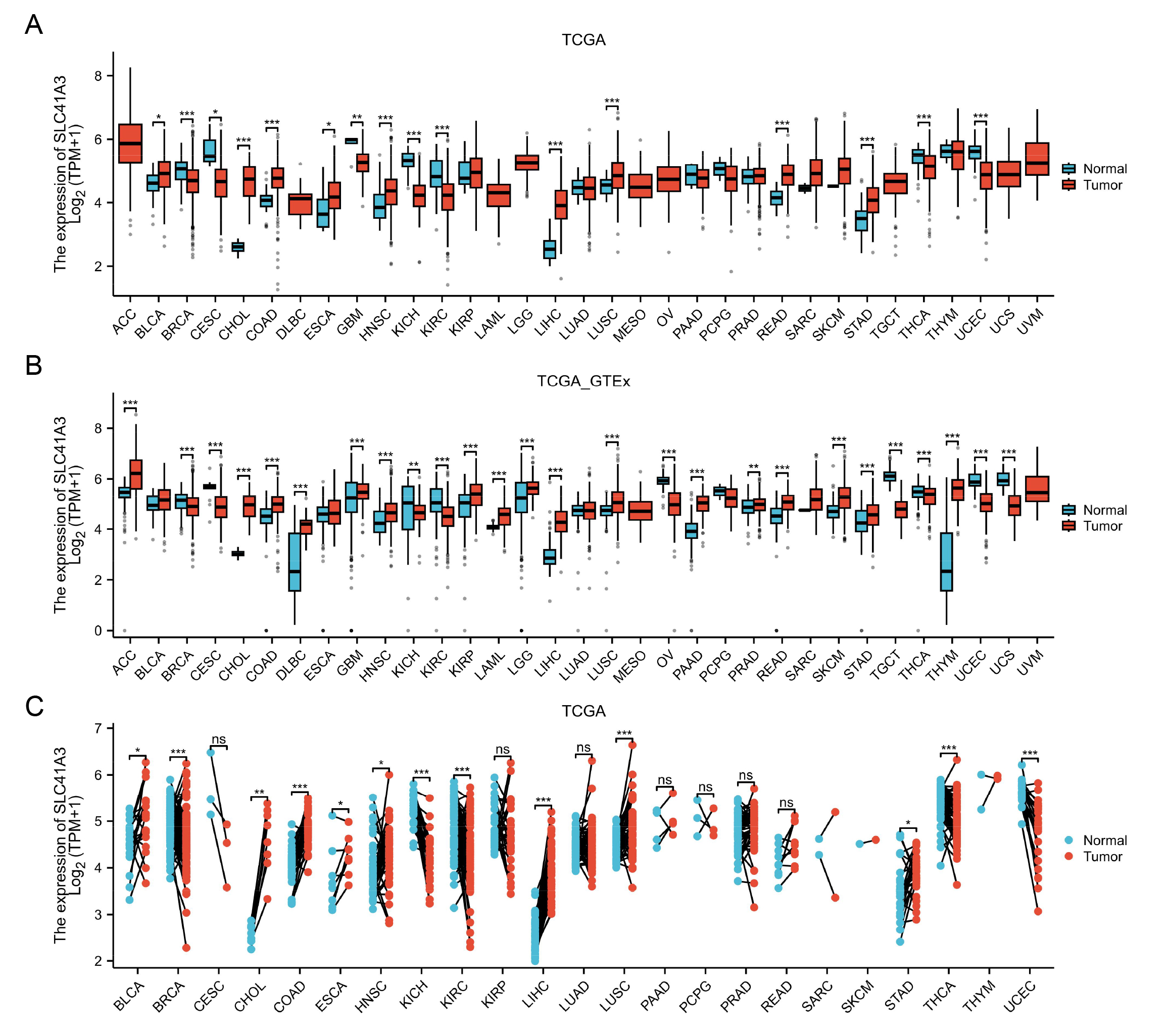


**Supplemental Figure 3. Pan-cancer expression analysis of SLC41A3.**

(A) Differential SLC41A3 expression patterns across various cancer types based on TCGA data. (B) Comparative analysis of SLC41A3 expression in human malignancies using the integrated TCGA and GTEx dataset. (C) Evaluation of SLC41A3 expression in tumor tissues versus matched adjacent normal tissues. ns *P* >0.05, * *P* <0.05, ** *P* <0.01, *** *P* <0.001.


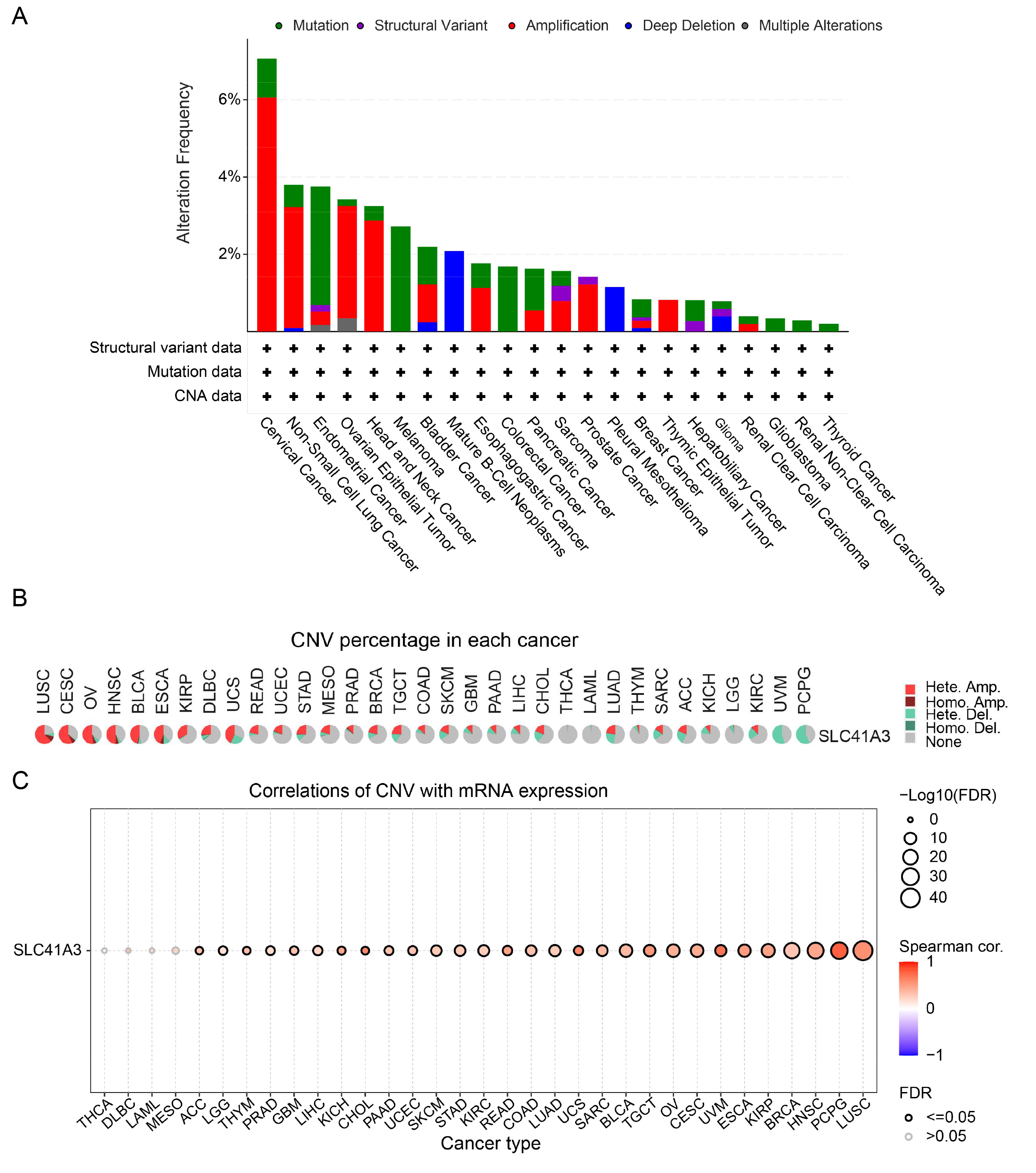


**Supplemental Figure 4. Mutation analysis of SLC41A3 across pan-cancer.**

(A) Distribution of SLC41A3 mutation types across various malignancies. (B) Pan-cancer analysis of the frequency of SLC41A3 copy number variation (CNV). (C) Relationship between SLC41A3 copy number alterations and their corresponding mRNA expression levels.


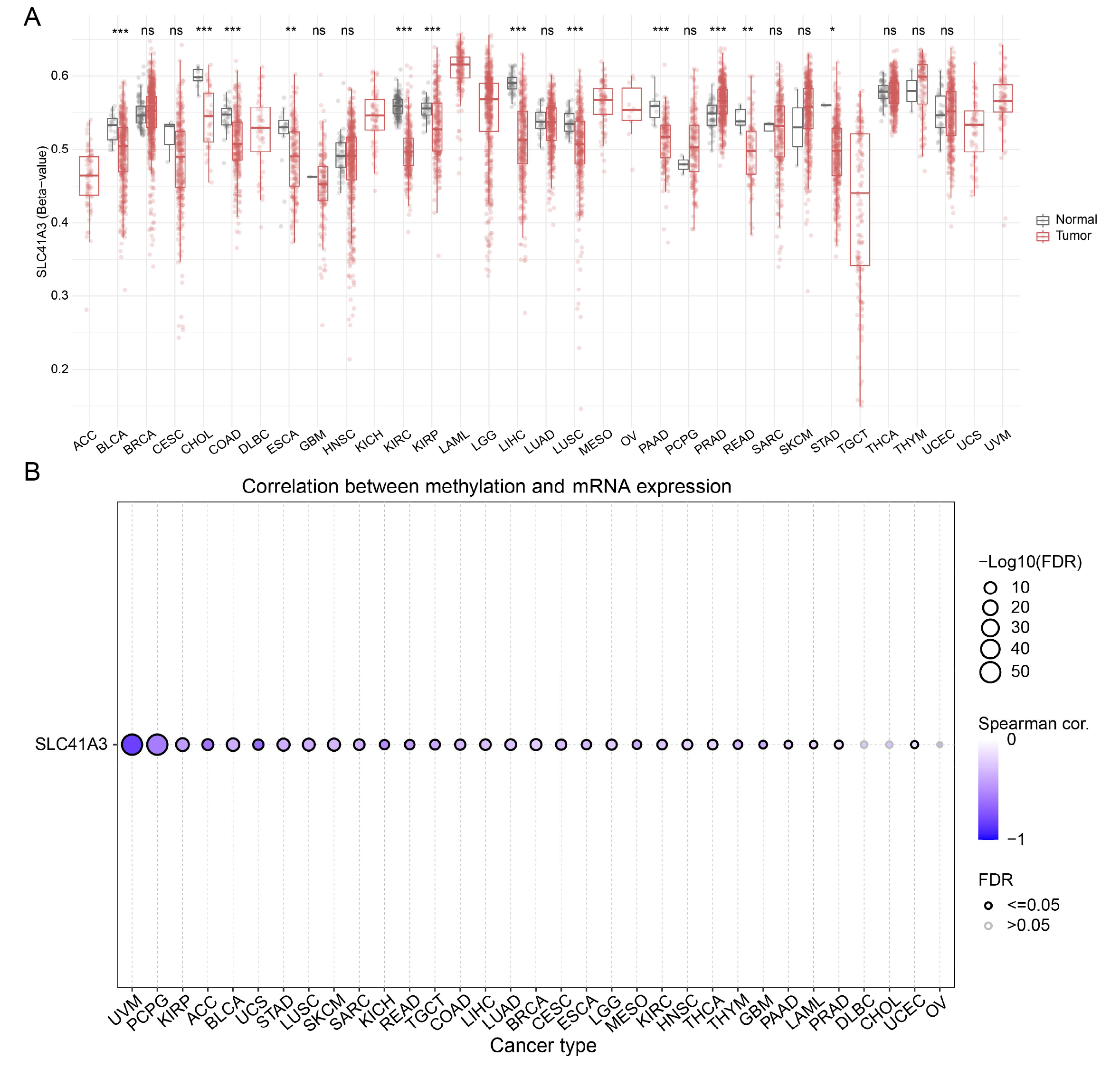


**Supplemental Figure 5. Comprehensive DNA methylation analysis of SLC41A3 across pan-cancer.**

(A) DNA methylation profiles of SLC41A3 across various cancer types. (B) Association between SLC41A3 expression levels and its promoter methylation status. ns *P* >0.05, * *P* <0.05, ** *P* < 0.01, *** *P* < 0.001.


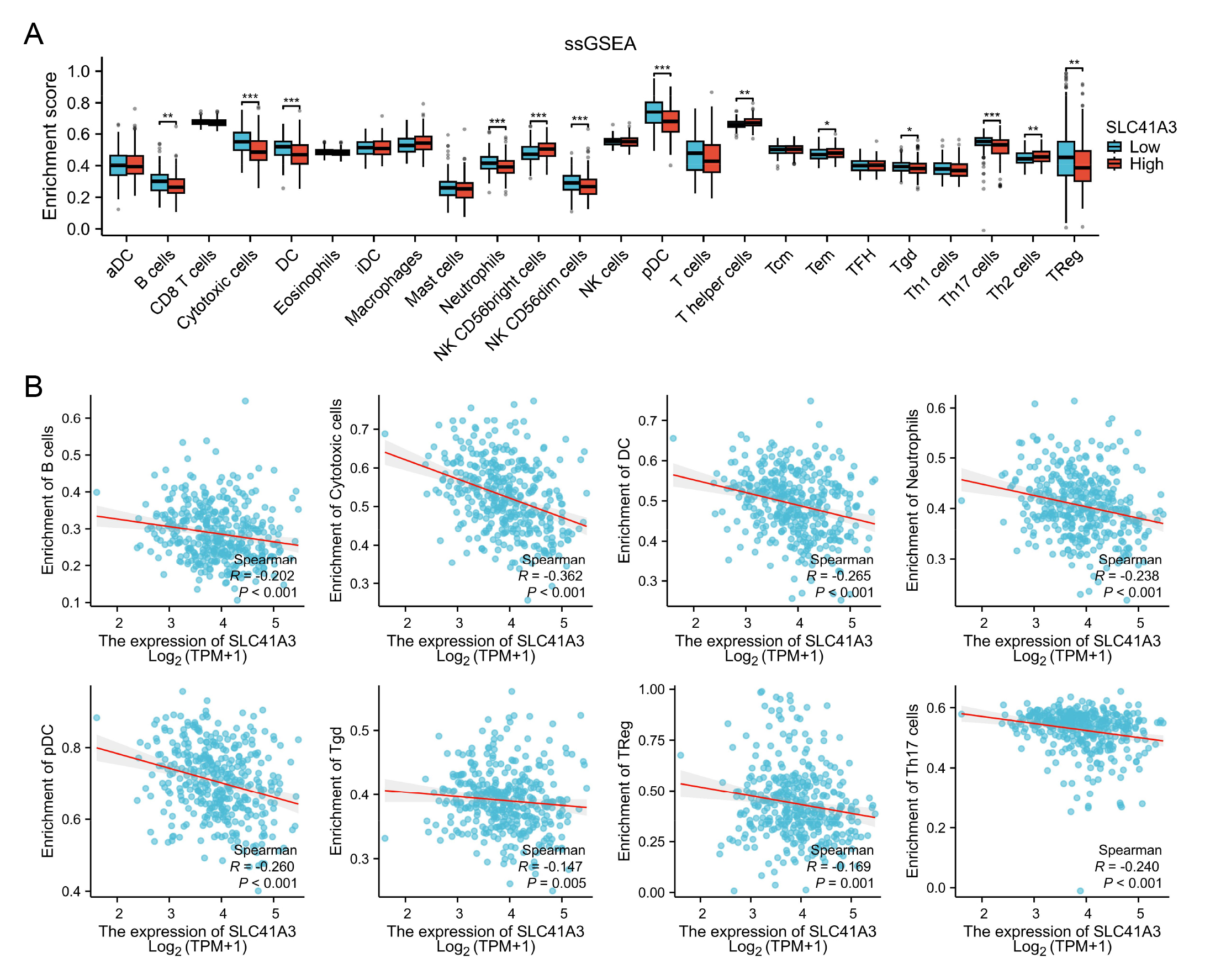


**Supplemental Figure 6. Relationship between immune infiltration and SLC41A3 expression in HCC.**

(A) Comparative analysis of immune cell infiltration between the SLC41A3 high-expression and low-expression groups from the TCGA-LIHC cohort, as assessed by ssGSEA. (B) Correlation analysis between SLC41A3 expression and signature scores for B cells, T helper cells, and central/effector memory T cells (Tcm/Tem). * *P* <0.05, ** *P* <0.01, *** *P* <0.001.


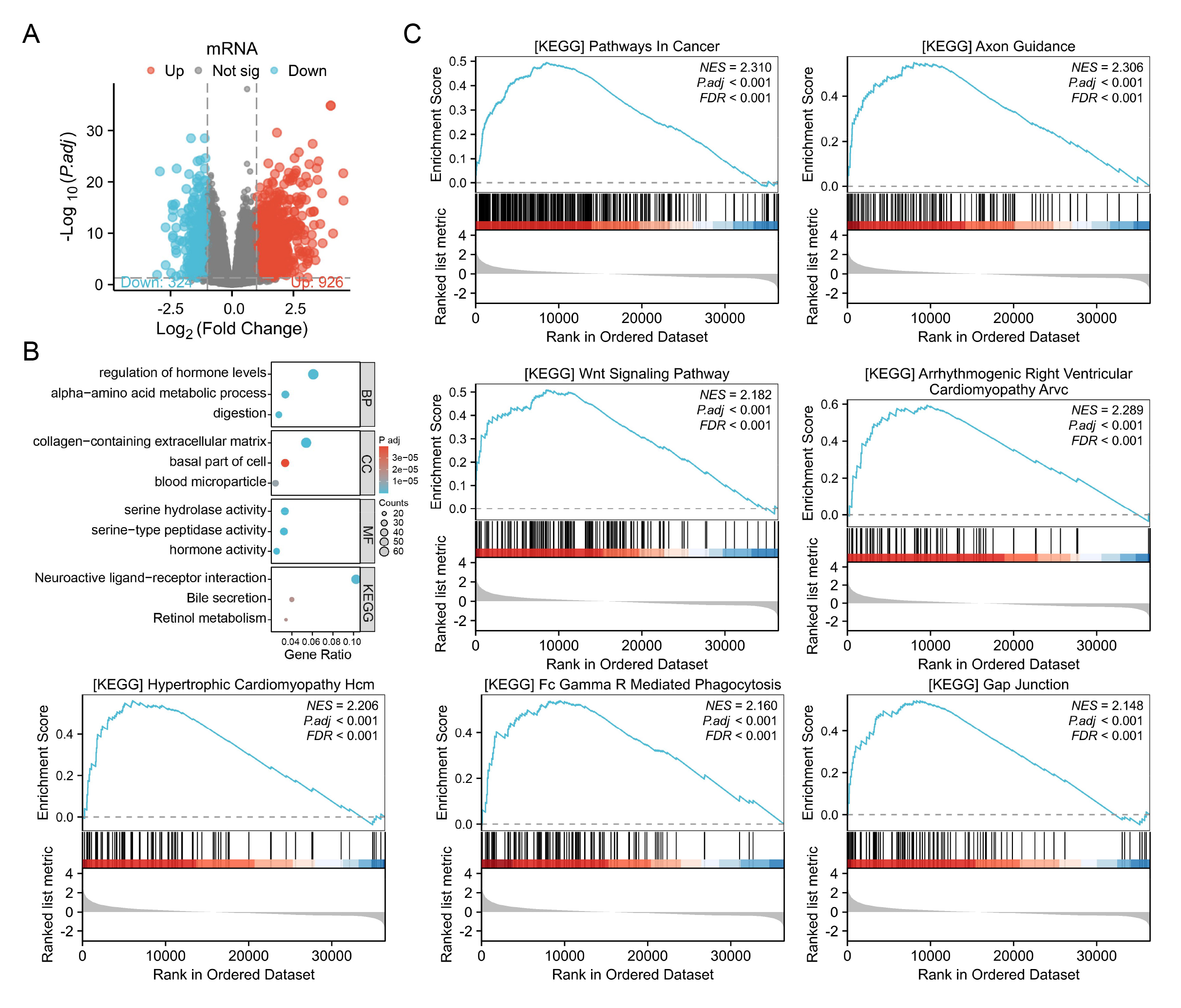


**Supplemental Figure 7. Identification of differentially expressed genes stratified by SLC41A3 expression levels in HCC.**

(A) An illustration of the mRNA expression differences between cohorts stratified by high versus low SLC41A3 levels is presented as a volcano plot. (B-C) Enrichment assessments, including KEGG pathway, Gene Ontology (GO), and Gene Set Enrichment Analysis (GSEA), were performed on the differentially expressed genes derived from the SLC41A3-based stratification.
